# Age-dependent ataxia and neurodegeneration caused by an αII spectrin mutation with impaired regulation of its calpain sensitivity

**DOI:** 10.1038/s41598-021-86470-1

**Published:** 2021-03-31

**Authors:** Arkadiusz Miazek, Michał Zalas, Joanna Skrzymowska, Bryan A. Bogin, Krzysztof Grzymajło, Tomasz M. Goszczynski, Zachary A. Levine, Jon S. Morrow, Michael C. Stankewich

**Affiliations:** 1 Department of Tumor Immunology, Hirszfeld Institute of Immunology and Experimental Therapy, Polish Academy of Sciences, Weigla 12, 53-114 Wrocław, Poland; 2 Department of Molecular Biophysics and Biochemistry, Yale University, New Haven, CT USA; 3 Department of Biochemistry and Molecular Biology, Wroclaw University of Environmental and Life Sciences, Norwida 31, 50-375 Wrocław, Poland; 4 Department of Pathology, Yale University School of Medicine, 310 Cedar Street, LH108, New Haven, CT 06520 USA; 5 Department of Molecular, Cellular, and Developmental Biology, Yale University, New Haven, CT USA

**Keywords:** Alzheimer's disease, Neurodegeneration, Cellular neuroscience, Cytoskeleton, Molecular modelling

## Abstract

The neuronal membrane-associated periodic spectrin skeleton (MPS) contributes to neuronal development, remodeling, and organization. Post-translational modifications impinge on spectrin, the major component of the MPS, but their role remains poorly understood. One modification targeting spectrin is cleavage by calpains, a family of calcium-activated proteases. Spectrin cleavage is regulated by activated calpain, but also by the calcium-dependent binding of calmodulin (CaM) to spectrin. The physiologic significance of this balance between calpain activation and substrate-level regulation of spectrin cleavage is unknown. We report a strain of C57BL/6J mice harboring a single αII spectrin point mutation (Sptan1 c.3293G > A:p.R1098Q) with reduced CaM affinity and intrinsically enhanced sensitivity to calpain proteolysis. Homozygotes are embryonic lethal. Newborn heterozygotes of either gender appear normal, but soon develop a progressive ataxia characterized biochemically by accelerated calpain-mediated spectrin cleavage and morphologically by disruption of axonal and dendritic integrity and global neurodegeneration. Molecular modeling predicts unconstrained exposure of the mutant spectrin’s calpain-cleavage site. These results reveal the critical importance of substrate-level regulation of spectrin cleavage for the maintenance of neuronal integrity. Given that excessive activation of calpain proteases is a common feature of neurodegenerative disease and traumatic encephalopathy, we propose that damage to the spectrin MPS may contribute to the neuropathology of many disorders.

Neurodegenerative disorders such as Alzheimer’s dementia, Parkinson’s disease, traumatic encephalopathy, and hereditary ataxias exhibit slowly-progressive neuronal death, neuroinflammation, and a notable loss in proteostasis. Inappropriate activation of calpains is one event postulated to accompany neurodegeneration^1,2^. Calpains regulate many cellular processes including neurite outgrowth, synaptic remodeling, autophagy control, learning and memory. With age or injury calpain activity in the brain naturally increases, with consequences on amyloidogenic APP processing, phospho-tau generation, autophagy, signal transduction, and cytoskeletal remodeling^1,3,4^. The introduction of pharmacological calpain inhibitors^5^ or over-expression of the natural calpain inhibitor calpastatin^6^ prevents or reduces neurodegeneration in murine models, supporting calpain’s etiologic role. Calpains, however, have many targets. Which targets are most consequential in promoting neurodegeneration remains unknown.

One calpain target that has long been recognized based on its characteristic break-down products (sBDP’s) is αII spectrin^7^. This spectrin is primarily cleaved by calpain at a single Y_1176_-G_1177_ site^8,9^, a site in close proximity to spectrin’s calmodulin binding domain^8^. Spectrin cleavage correlates with neuronal excitability, glutamate neurotoxicity, and synaptic plasticity^9,10^. Spectrin sBDP’s often appear in the brains of Alzheimer’s disease patients, along with classical hallmarks such as insoluble aβ plaques and neurofibrillary tangles^11,12^. Spectrin’s susceptibility to cleavage is regulated by both calmodulin(CaM)^9,13,14^ and by Y_1176_ phosphorylation^15,16^. These molecular regulators are of significance for proper neuronal function, since cleavage of αII-spectrin at Y_1176_ reduces its ability to cross-link actin filaments, a key feature of the neuronal spectrin skeleton^17^, and exposes its normally-protected paired beta subunit to calpain attack^18^. These subtle interactions highlight the diverse role of spectrin in neuronal ecology and the central role of cellular scaffolds in disease etiology.

Recent studies reveal details of a neuronal membrane-associated periodic skeleton (MPS) composed of spectrin, actin, and associated adapter proteins^19^. Beyond presumably stabilizing the neuronal membrane, the MPS appears to be integral to neuronal development and homeostasis. Its functions include the mediation of transmembrane signaling^20^, the modulation of both retrograde and anterograde signals that control axonal remodeling and degeneration^21^, and the control of conductance channels at the nodes of Ranvier^22^. The activation of extracellular signal-regulated kinases (ERK’s) can also activate calpain to locally remodel the MPS^20^. Given this central role of spectrin, it is thus not surprising that defects in spectrin cause neurologic pathology. Mice lacking αII spectrin are embryonic lethal with cardiac and nervous system malformations^23^. Human mutations in αII spectrin (*SPTAN1)* link to early infantile epileptic encephalopathy (EIEE or West Syndrome)^24^ and other hereditary neuropathies. Defects in the beta spectrins lead to other forms of neurologic disease.

We herein identify a novel αII spectrin R1098Q variant C57BL/6J mouse strain with an autosomal dominant ataxic and neurodegenerative phenotype. The R1098Q spectrin exhibits reduced affinity for calmodulin and undergoes accentuated calpain-mediated cleavage. Molecular models and solution biophysical measurements offer a plausible explanation of how this single point mutation in spectrin selectively impacts the intrinsic sensitivity of the protein to calpain attack, while leaving spectrin’s other functions unaltered. It appears that mutations at R1098 disrupt salt-bridging between two sequential repeat subunits, modifying the CaM-binding site and constitutively exposing the preferred physiologic site of calpain cleavage to attack. These results indicate that a delicate balance must exist between the physiologic activation of calpain, and substrate-level control of spectrin cleavage. Disrupting this balance is sufficient to induce a progressive age-dependent ataxia with neurodegeneration. It is proposed that damage to the spectrin based MPS by excessive calpain activity may be a consequential event contributing to the pathology of many ataxias and neurodegenerative disorders.

## Results

### Mice with the Sptan1 (αII spectrin) R1098Q mutation develop progressive ataxia with tremors

We identified a C57BL/6J mouse with spontaneous onset of an unsteady gait. This mouse served as the founder strain and was bred back onto the WT C57BL/6J line. Affected animals began to develop poor coordination within months after birth, and the ataxia was fully developed after six to nine months (supplemental movie M1). This was quantitatively documented by rotarod evaluation and related measures that indicate a steady decline in balance and motor control that becomes detectable within 4–10 weeks of age, depending upon the severity of motor testing (supplemental Fig. S1). The ataxia progresses as the mice age, and breeds as a Medelian-dominant trait. Average litter size of heterozygous crosses are reduced by ≈ 25%, consistent with homozygote embryonic lethality. Embryos die by day eighteen (E18) with severe craniofacial and vascular defects (supplemental Fig. S2). The phenotype shows insignificant gender differences (heterozygotes 44% female, 56% male); adult animals are fertile without excess mortality up to the length of our experiments (≈ 2 years). Although not measured, it seems that ataxic mice when handled are typically more aggressive and have episodes of tremor and seizure, features reminiscent of ataxic mice with βIII spectrin deficiency^25^. Other (non-nervous) tissues appear to function normally.

Whole exome sequencing (WES) (data deposited in the NCBI sequence read archive as BioProject: ID 682493, https://www.ncbi.nlm.nih.gov/sra/PRJNA682493 ) comparing unaffected and affected littermates revealed non-synonymous genomic variation in 34 candidate genes spanning 17 chromosomes (supplemental Table S1; VCF file of variants identified in the C57BL/6J littermates available from authors upon request). Pathway prediction and PCR analysis established that variation in Sptan1 ((NM 001177667): c.3293G > A:p.R1098Q) was the most likely cause of the phenotype. Analysis of this locus in 24 embryos and 190 adult mice exhibited a perfect (100%) correlation between the phenotype and the Sptan1 c.3293G > A; R1098Q genotype, yielding a combinatorial probability of < 1 × 10^−34^ that the phenotype could be caused by any of the other 33 genes with non-synonymous changes that were identified in the littermates.

**Figure 1:**
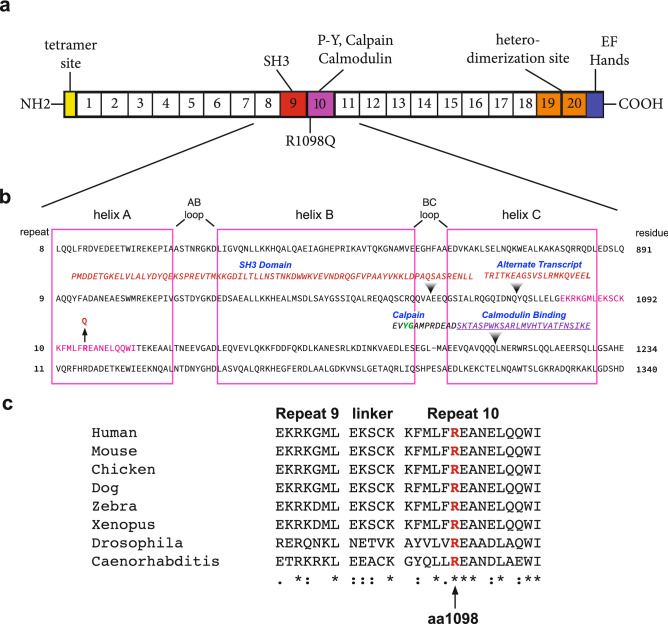
Relationship of the R1098Q variant to αII spectrin structure. (**a**) Schematic representation of αII spectrin with 20 complete homologous repeats each ≈ 106 residues and the location of selected functional domains that interrupt the repeat motif. The 9th and 10th repeats (red and purple respectively) harbor an inserted SH3 domain, a variably appearing alternative transcript insertion, and specialized sequences encompassing the site of CaM binding and the site Y1176 and G1177 that is preferentially cleaved by calpain. (**b**) Sequence of repeats 8–11 aligned by homology. The boxes denote the boundaries of each of the three α-helices (A–C) of the canonical repeat unit; these are separated by AB and BC loops and inter-unit linkers. The sequences representing the SH3 domain and the alternative transcript inserted into repeat 9 and the calpain and CaM interaction sites in repeat 10 are indicated. The arrow marks the site of the R1098Q mutation near the start of the 10^th^ repeat. The YG site of preferred calpain cleavage is colored green. (**c**) Clustal alignment of residues flanking the R1098Q mutation. This locus, and the flanking region, is almost perfectly conserved across diverse species. Residues fully conserved are denoted with asterisks (*); residues strongly conserved with a score greater than 0.5 on the PAM250 matrix are designated with a colon (:). Weakly similar residues are denoted with a period (.).

The non-synonymous point mutation in Sptan1 converts a highly conserved arginine at residue 1098 to a glutamine. This mutation is near the start of the A helix of spectrin’s tenth triple helical repeat (Fig. 1). This repeat also contains the sites of calpain and caspase cleavage and spectrin’s CaM binding site^26^. (When numbering the repeats of αII spectrin, we follow a convention that does not ascribe repeat sequence to non-homologous specialized domains^27^. Using this framework, the calmodulin binding is in the 10^th^, and not the 11^th^, repeat unit of αII spectrin).

### Progressive age-dependent cerebellar degeneration in R1098Q mice

**Figure 2:**
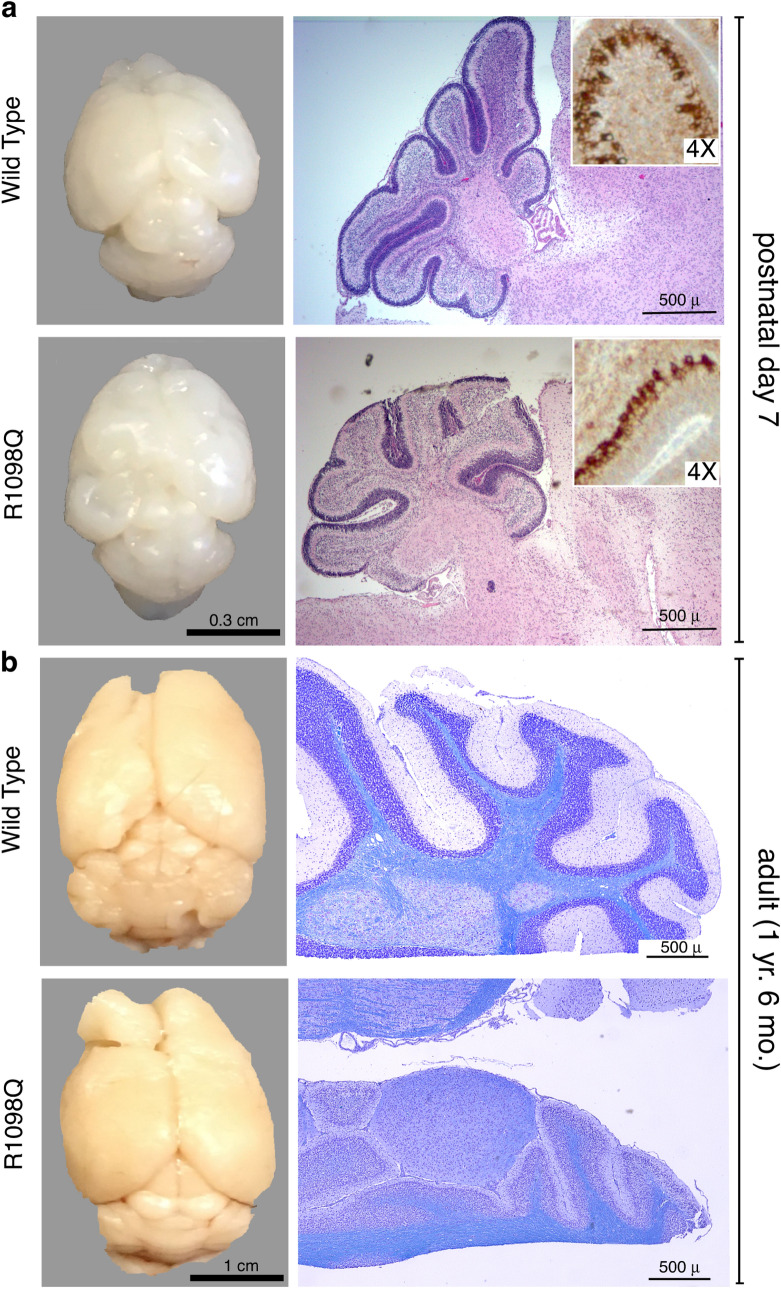
Whole brain images and cerebellum histology. (**a**) WT and heterozygous R1098Q mice brains are indistinguishable at birth. By P7, subtle gross and histologic abnormalities begin to emerge in the brains of the R1098Q heterozygotes. These include nearly imperceptible defects in their cerebellar folia and cerebral convolutions. However, at P7 Purkinje cells are abundant and their morphology is intact, excluding developmental failure due to the heterozygous mutation. This is most apparent when immunostained for βIII spectrin that highlights the PC soma and dendrites (**insert**, shown at 4 × magnification of the H&E stained section; also see supplemental Fig. S3 for enlarged image of the P7 cerebellum). (**b**) Older R1098Q heterozygote mice (1.5 years) exhibit profound atrophy of the cerebellum, correlating with near total collapse of the molecular layer and loss of Purkinje cells. Some cortical loss can also be perceived. These gradual morphologic changes correlate with the onset of the progressive ataxia apparent in the R1098Q mice, a phenotype that intensifies with age.

**Figure 3:**
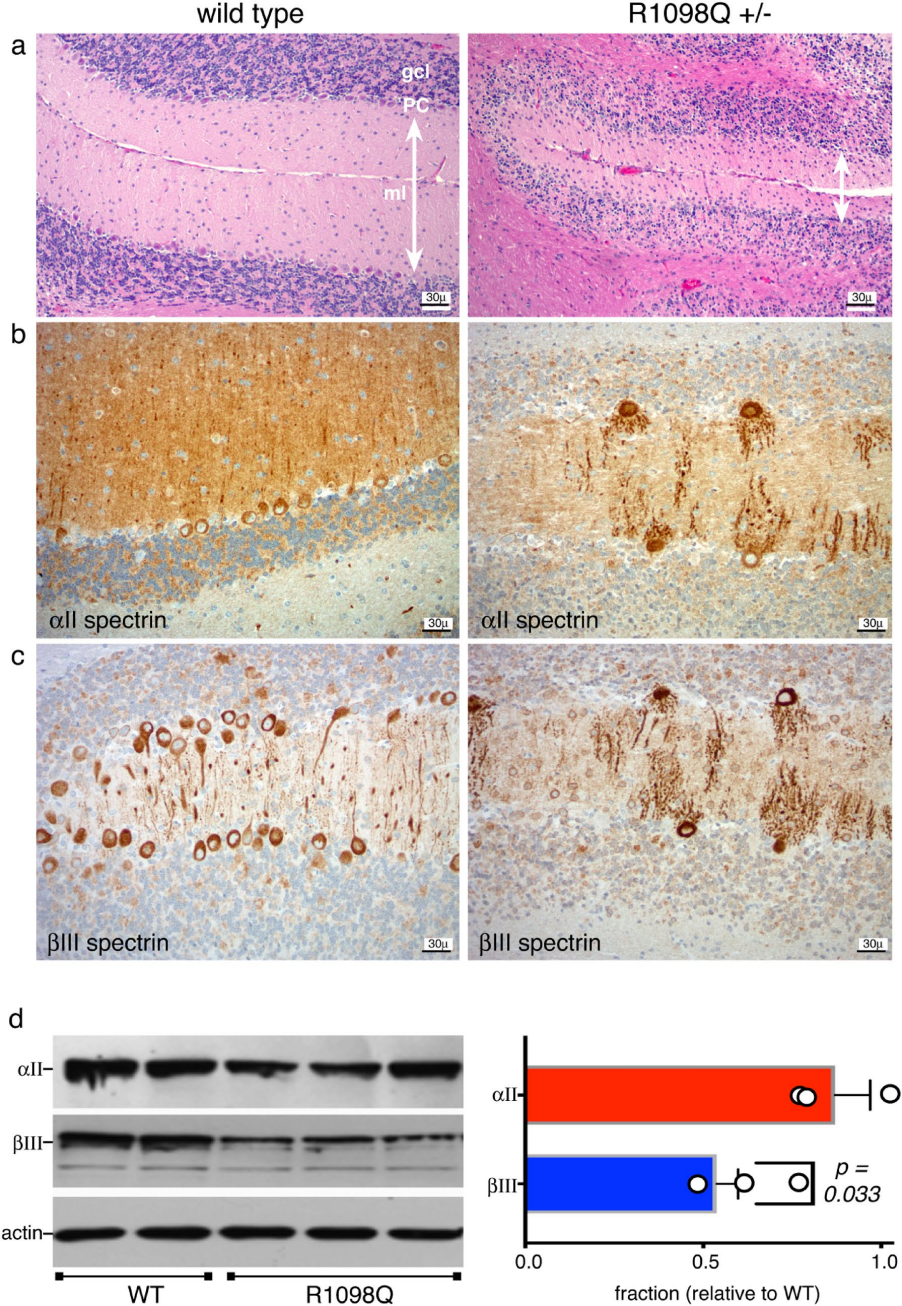
Cerebellar pathology in R1098Q heterozygous mice. (**a**) Hematoxylin and eosin (H&E) stained sections of cerebellum of 26 week old WT and heterozygous R1098Q mice. Most noticeable is the ≈ 50% loss in molecular layer width (double headed arrows) and extensive loss of cells in the Purkinje layer.. These changes are quantified in Supplemental Fig. S7. There may also be some loss of PC’s in the granular layer as well, but the disruption of layer organization in the hets made defining the boundaries of the granular layer problematic and rendered quantitation unreliable. (**b**) Immunostain for αII spectrin reveals its preservation in all cerebellar layers, and highlights the loss of PCs and the severe disruption and fragmentation of PC dendrites in the R1098Q mice. (**c**) Immunostain for βIII spectrin. This spectrin is primarily (although not exclusively) expressed in PCs in the cerebellum. Its pattern is the same in PC’s as that of αII spectrin, highlighting the major distortions and fragmentation of PC dendritic architecture. (**d**) Western blot of adult cerebellum for αII and βIII spectrin quantifying the global and selective PC loss in heterozygotes, expressed as a ratio relative to the WT levels. Results from two WT and three R1098Q heterozygotes are shown. While there is no significant reduction in αII spectrin levels (which is expressed in all cells), the βIII spectrin level is reduced to 64 ± 7% of the WT level (*p* = 0.033), reflecting the selective loss and vulnerability of PC’s to the R1098Q mutation. (Full length blots of the gels used for quantitation are presented in supplemental Fig. S6). Data analyzed by single tail, 2-sample homosedastic T-test. N = 2 for WT; 3 for het.

The in-utero development and death of homozygous R1098Q animals phenocopies in most respects animals with complete loss of αII spectrin^23^. This is not the case for heterozygous Sptan1 R1098Q animals. While animals with single WT spectrin allele and a null second allele (Sptan1^(+/−)^) are phenotypically normal, the presence of an allele encoding the R1098Q spectrin variant (in lieu of a null allele) is toxic. Heterozygous R1098Q neonates at postnatal day 7 (P7) display subtle gross anomalies in their brain, including foliation of the cerebellum and convolutions in the cortex (Fig. 2, contrast with supplemental Fig. S2). By adulthood, the degree of cerebellar degeneration becomes profound, and correlates with the progressive decline in coordinated movement (supplemental Fig. S1). Histologically, alterations in the neonatal P7 brain are subtle and largely unapparent. Purkinje cells at this early age appear well developed and abundant, without obvious disruption of their dendrites or number. This is particularly apparent after immunostaining for βIII spectrin, a marker of PC’s in the cerebellum (Fig. 2a, inset; Fig. 3, supplemental Figs. S3, S7). By 26 weeks of age however, there are significant defects in molecular layer width (reduced by over 50%) and in neuronal organization and number (Fig. 2b, supplemental figure S7). Purkinje cells (PC) are reduced by over 80% (Fig. 3a, supplemental figure S7). While the overall expression pattern of αII spectrin (which is present in all cells of the cerebellum) and βIII spectrin (confined to PC soma and dendrites) is preserved, the selective loss of PC’s in the mutant animals is reflected by the global loss in the adult cerebellum of βIII spectrin relative to αII spectrin (Fig. 3b-d). A profound disruption of PC dendrite morphology and its planar orientation is also apparent. Whereas normal PC apical dendrites visualized in a coronal plane appear thin with limited branching and span the molecular layer, the PC dendrites in adult R1098Q heterozygotes are thickened, fragmented, highly branched, and foreshortened.

**Figure 4:**
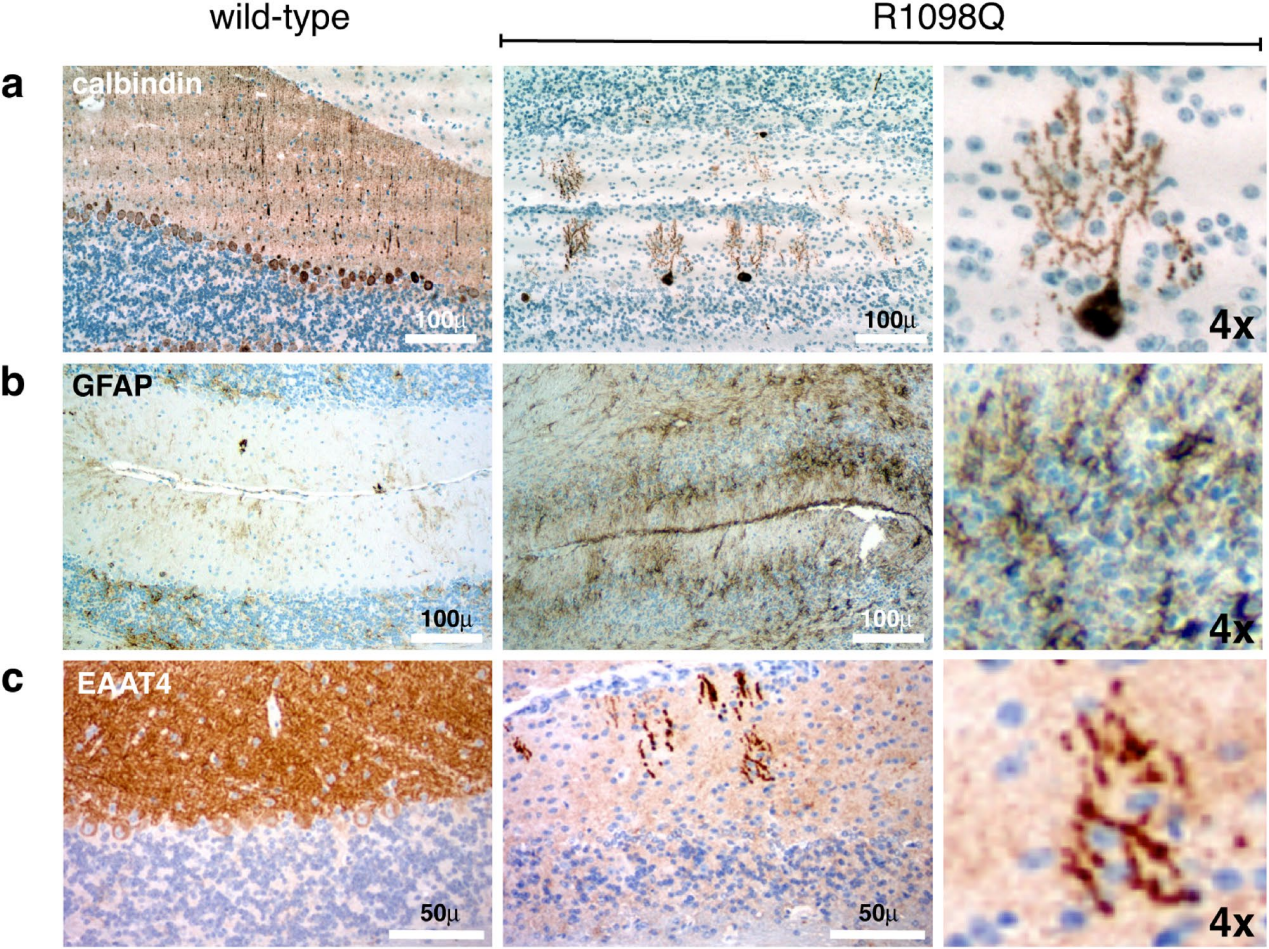
Evidence of cerebellar degeneration and gliosis. We used immunostains with markers of degeneration, reactive gliosis, and synaptic integrity to further characterize the cerebellar changes in 1.5 yo R1098Q heterozygous mice. Images in the right-most panel of each row are presented at 4 × magnification relative to the other images. (**a**) Calbindin, a marker of PCs, highlights their dramatic loss and their excessively branched, segmented, and coarse dendrites. (**b**) Glial fibrillary acidic protein (GFAP), a marker of reactive gliosis and an indirect marker of neuroinflammation, reveals a marked increase in both the molecular and granular cell layers. Astrocytes and their processes infiltrate all cerebellar layers. (**c**) EAAT4, a glutamate transporter that binds spectrin at Purkinje cell PSDs, is nearly absent in the R1098Q mice, and what remains is concentrated in the swollen dendritic shafts of PCs.

We next searched for molecular markers of neurodegeneration and neuroinflammation. The dramatic loss of PC’s and their aberrant dendritic pattern in 26 week to 1.5 year old mice is illustrated by immunostains with calbindin, a PC cell marker (Fig. 4a, supplemental Fig. S7). Reactive astrogliosis typically accompanies neuroinflammation and CNS pathology; its hallmark is up-regulation of glial fibrillary acidic protein (GFAP)^28^. GFAP levels are elevated substantially in the cerebellum R1098Q mice by 26 weeks of age, and continue to rise as the mice age (Fig. 4b, supplemental Fig. S7). Finally, we were interested in the fate of the excitatory amino acid transporter 4 (EAAT4), a major spectrin ligand that is depleted from the PC synapse in spectrin-dependent ataxias^25,29^. Consonant with the abnormalities observed in the PC’s, EAAT4 is nearly absent in the 1.5 year old R1098Q mice, and what is present is confined to coarse aggregates within the thickened and shortened dendritic shafts of PC’s (Fig. 4c).

### The cerebellum of R1098Q mice display ultrastructure hallmarks of degeneration

**Figure 5:**
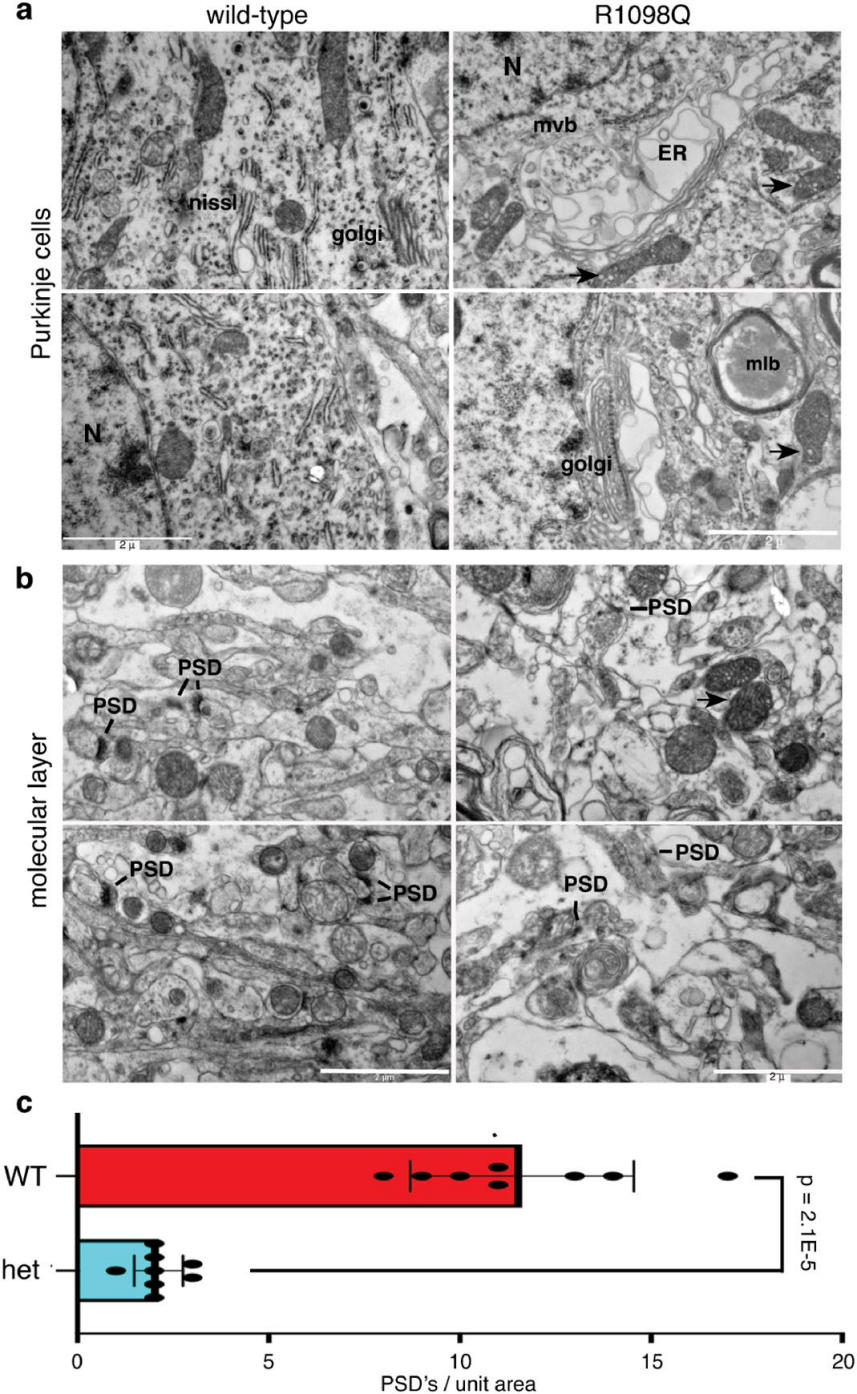
EM analysis reveals degenerative changes in R1098Q PCs and the molecular layer. (**a**) The PC’s of R1098Q 1.5 yo mice exhibit massively dilated organelle structures that appear to arise from Golgi and ER, along with dispersal of Nissl substance. Many multivesicular bodies (mvb) and multilamellar bodies (mlb) are present, presumably derived from autophagocytic vacuoles. Mitochondia (arrows) also frequently show internal vesiculation.(**b**) Within the cerebellar molecular layer of the heterozygous animals, dendrites are swollen and post synaptic densities (PSD) are attenuated and sparse. (**c**) The PSD’s identified in eight separate EM images of WT mouse and eight images from a heterozygous R1098Q mouse were counted. There is an approximately four-fold reduction in PSD’s in the heterozygous 1.5 yo mouse. The variation in the replicates comparing these two mice were evaluated by paired single tail T-test.

Electron microscopic analysis of 1.5 year old R1098Q mice reveals cellular and subcellular changes reflective of neurodegeneration^30^. Purkinje cells of heterozygous R1098Q mice display markedly distended membrane compartments that appear to be in continuity with Golgi and ER profiles (Fig. 5a). Nissl substance is poorly organized, and multivesicular (msb) and multilamellar bodies (mlb) are prominent. Many mitochondria show internal vesiculation (Fig. 5, arrows). These changes are similar to those seen in PC’s with complete βIII spectrin deletion^25, 31^.

The cerebellar molecular layer shows many of these same changes (Fig. 5b). The number of post-synaptic densities (PSDs) is markedly reduced compared to WT mice (Fig. 5c), and appear attenuated. The PSDs that remain often reside on dendritic shafts or the soma rather than spines, a feature also observed in cultured neurons that have lost βIII spectrin^32^.

### The neocortex and hippocampus of R1098Q mice display neuronal loss and reactive gliosis

**Figure 6:**
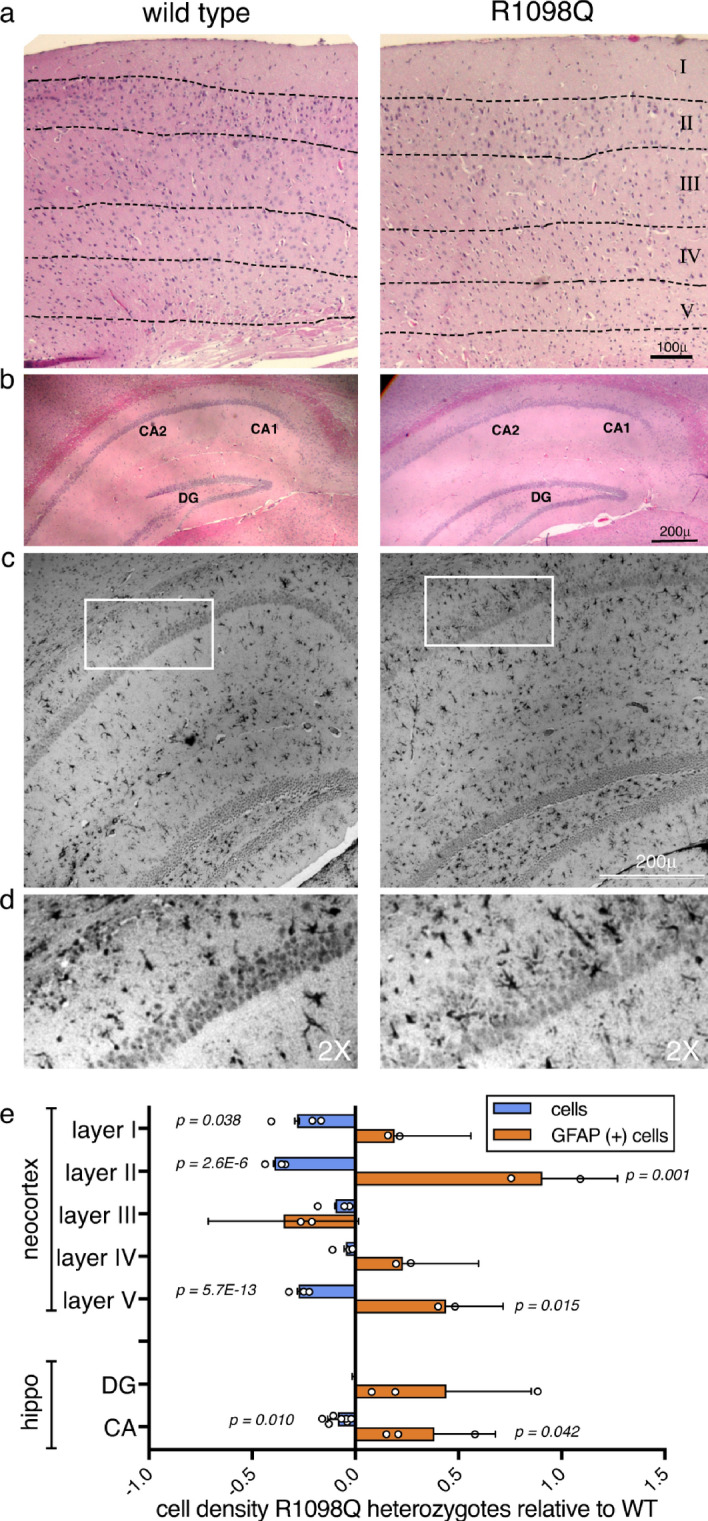
Degeneration of the neocortex and hippocampus in adult R1098Q mice. While not as dramatic as in the cerebellum, there is significant astrogliosis and neuronal loss in the cerebrum of 1.5 year old mice. (**a**) H&E stained neocortex exhibiting neuronal loss. When the cells per unit area and their GFAP staining were counted (below), losses and enhanced GFAP positive cells were significant in layers I, II and V. (**b**) H&E stained hippocampus. As in the neocortex, there is cell loss particularly in the CA1/CA2 regions. (**c**) GFAP immunostaining of the hippocampus exhibiting widespread astrogliosis. The region outlined is depicted in greater detail in panel (**d**), where the infiltration of astroglia is apparent throughout the CA1/2 layers in the R1098Q animals. (**e**) Comparison of changes in cell and astroglial (GFAP positive) density in R1098Q heterozygotes relative to the WT adult brains. The average density of cells in cortical layer I, layer II, and V are significantly reduced, while there is significantly increased numbers of GFAP cells per unit area in layers II and V. The CA1/2 region also exhibits less dramatic but still reduced cell counts, with marked increase in GFAP positive cells. There is no significant change in cell density in the dentate gyrus (DG), and the increase in GFAP positivity in this region also did not reach significance. For this analysis, images from comparable regions of two WT and two heterozygous mice were each divided into equal area units (18 to 46 units depending on the size of the region under consideration), and the number of cells in each unit area counted by three independent observers. All data for WT and het were then each pooled, and the average cell densities of each region calculated and compared (WT vs. het). Significance was evaluated by paired single-tailed T-test.

While the dominant phenotype of the R1098Q mice is ataxia, given the widespread expression throughout the brain of αII spectrin, we examined the neocortex and hippocampus for evidence of more global neurodegenerative change in 1.5 year old animals. The neocortex exhibits significant loss of neurons/unit area in layers I, II and V (Fig. 6a). Conversely, the density of GFAP positive astrocytes was increased throughout the neocortex, indicating the presence of significant reactive gliosis in this region (Fig. 6e). We detected similar changes in the CA1-CA2 regions of the hippocampus (Fig. 6b). The number of pyramidal neurons was reduced and they were less tightly packed and were infiltrated heavily by GFAP positive astrocytes (Fig. 6c–e).

### The R1098Q spectrin mutation weakens calmodulin (CaM) binding and enhances its in-vivo proteolysis by calpain

**Figure 7:**
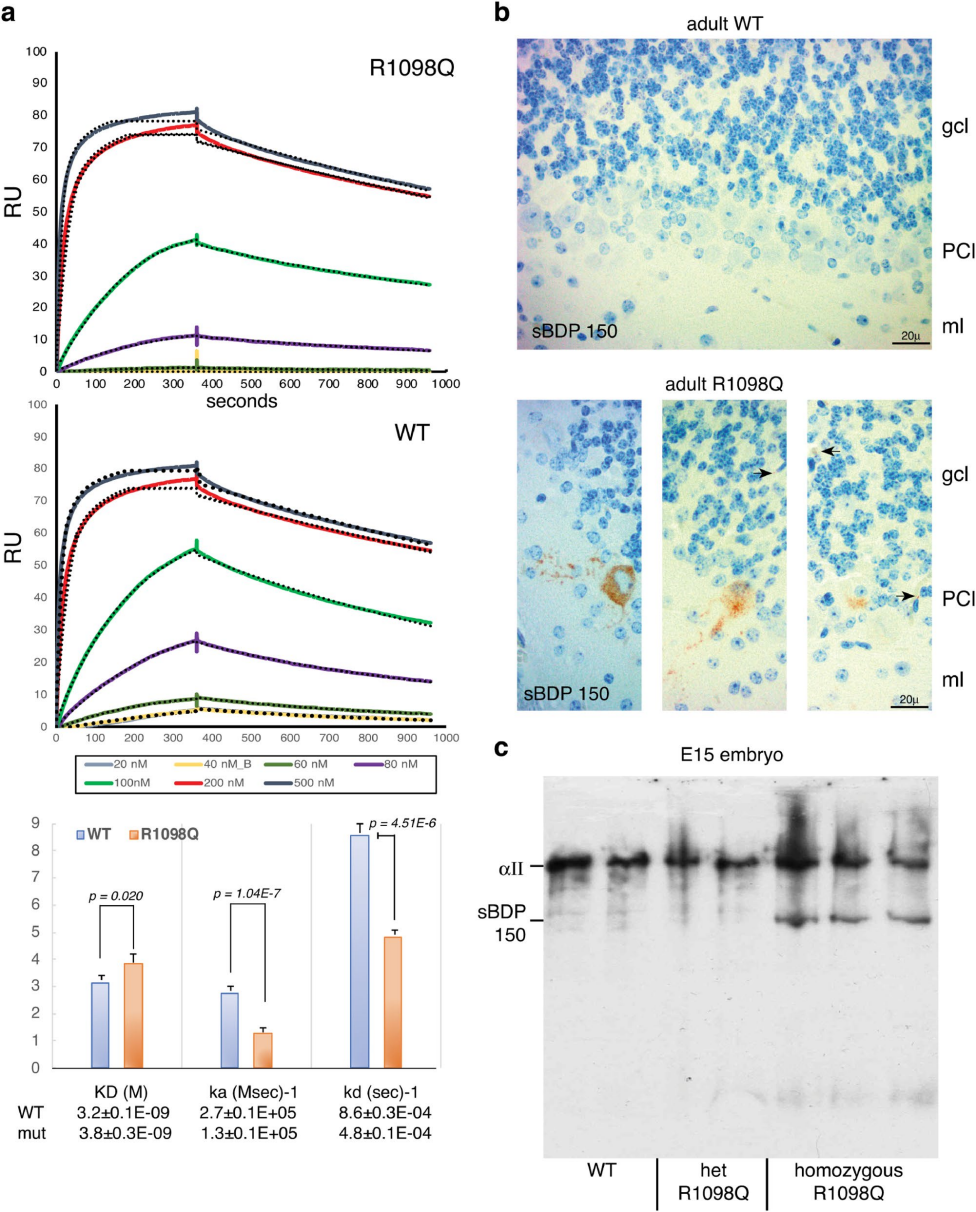
R1098Q spectrin exhibits reduced in vitro CaM affinity and enhanced in vivo calpain cleavage. (**a**) Surface plasmon resonance detected the binding of Ca^2+^/CaM to recombinant GST-spectrin peptides representing repeats 8–11 (as shown in Fig. 1). The concentration of CaM used for each curve is as indicated. The binding curves fit well to a 1:1 Langmuir binding model over the indicated range of ligand concentrations (dotted lines, generated by BIAevaluation 3.1, https://www.biacore.com/lifesciences/service/downloads/software_licenses/biaevaluation ). The derived rate constants for (ka) and (kd) and their confidence limits as reported by the BIAevaluation software were both reduced for the R1098Q peptide relative to the WT peptide. The derived KD for the mutant peptide of 3.8 ± 0.3E−09 represents a significant 19% reduction in its affinity for CaM. (**b**) To determine if spectrin was being excessively targeted by calpain in vivo, we examined the cerebellum of 26 week old mice with an antibody raised to the unique epitope created by calpain cleavage of spectrin at Y1176^9^. This antibody only detects calpain-cleaved spectrin. No staining was detected in the cerebellum of either of two WT animals. Conversely, their respective R1098Q heterozygous littermates (two animals) at 26 weeks of age displayed calpain-cleaved spectrin in 5–18% of their PC soma and throughout the apical dendrite. Cleavage product was also detected in the granular cell layer (arrows); the origin of this staining in the granular layer (whether cells or synapses with sBDPs) is undetermined. (**c**) Embryos (14.5 d) were harvested in-utero and analysed by Western blotting with anti-αII spectrin antibodies to gauge the level of spectrin proteolysis. While break-down was minimal or absent in both the WT and heterozygous embryos, a single sBDP at ≈150 kDa was prominent only in the homozygotes. This sBDP is the major product generated by calpain^9^. The characteristic cleavage fragment generated by caspase at ≈ 120 kDa during apoptosis^74^ was notably absent. These results indicate that the R1098Q spectrin is uniquely sensitive to calpain attack in the homozygous embryo (N = 2 WT; 2 het; and 3 homozygotes).

The proximity of αII spectrin’s tenth repeat unit to the sites of calpain cleavage and CaM binding led us to explore the impact of the mutation on these processes. The direct binding of calmodulin to a GST-fusion peptide representing the 9^th^-10^th^ repeat units of spectrin with or without the R1098Q mutation was measured in vitro by surface plasmon resonance (Fig. 7a). We find that both the association (K_a_)and disassociation (K_d_) of calmodulin binding to the mutant spectrin peptide is notably suppressed, yielding a net ≈19% drop in spectrin’s calcium-dependent affinity for CaM. We also looked for in vivo evidence of accelerated calpain degradation of αII spectrin (Fig. 7b). An antibody that specifically recognizes calpain-cleaved αII spectrin^9^ did not stain any PC’s in 26 week old animals (zero positive PC’s per 50 examined in each of two animals), but stained 5–18% of the PC’s in two R1098Q heterozygotes. Thus, by 26 weeks of age, the heterozygous animals already accumulate significant levels of calpain-cleaved spectrin. Alternatively, we examined E18 embryos harvested in-utero for evidence of sBDP’s (Fig. 7c). Using polyclonal antibodies to αII spectrin, we detect the parent protein along with its assorted degradation products. The calpain-cleavage specific proteolysis fragment of spectrin appears at ≈ 150 kDa. Importantly, caspase cleavage products of spectrin, as typically seen under conditions of apoptosis, appear at ≈ 120 kDa^9^. In the R1098Q homozygous embryos, there is significantly more calpain proteolysis of spectrin (≈ 150 kDa band) compared to either heterozygous or WT embryos. The absence of a band at ≈ 120 kDa indicates an absence of significant caspase cleavage. Collectively, these findings indicate that the R1098Q variant spectrin has impaired CaM binding and undergoes enhanced proteolysis by calpain in vivo.

### Molecular models corroborate reduced spectrin-CaM interactions and enhanced sensitivity to calpain proteolysis

**Figure 8:**
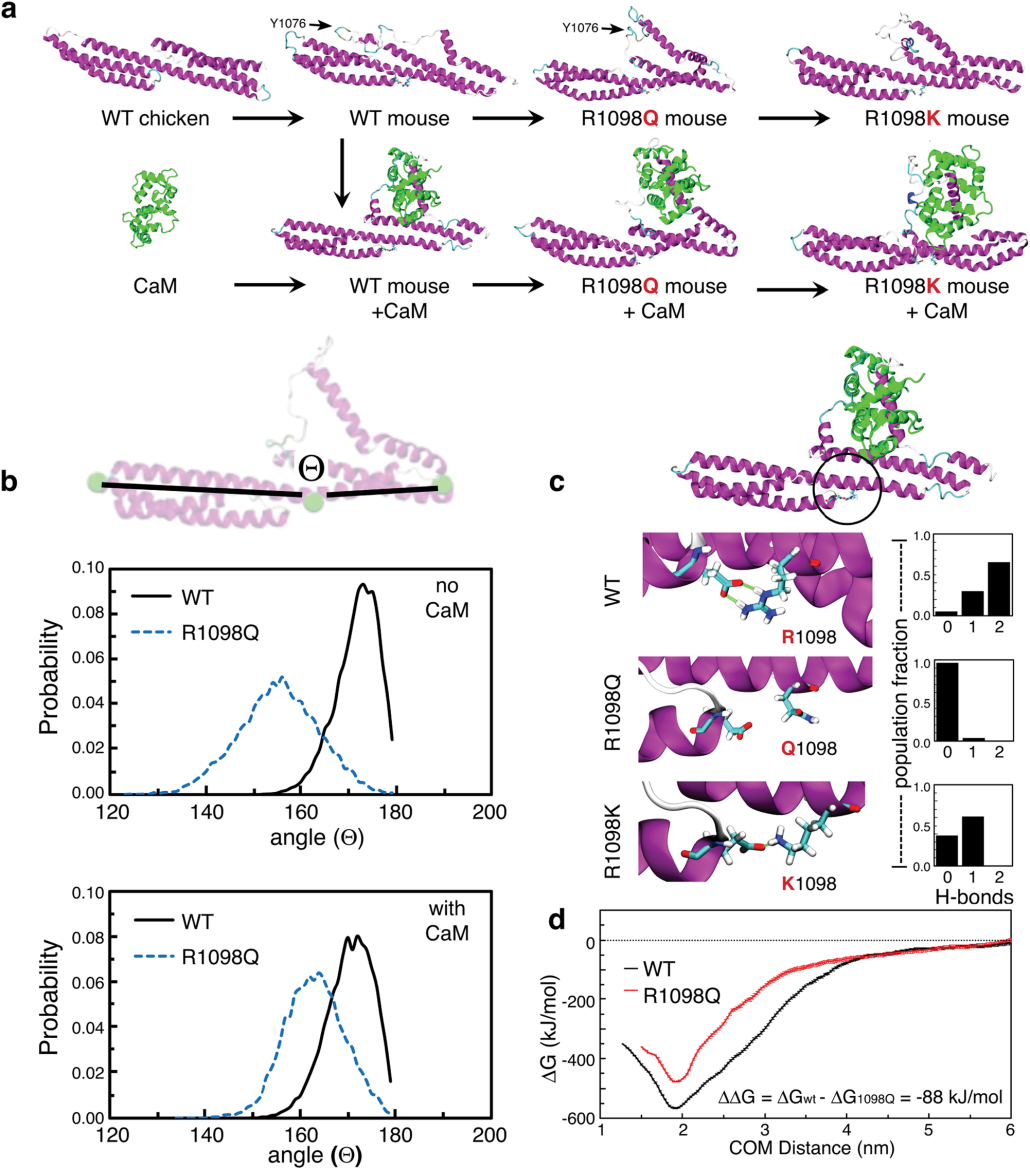
Molecular dynamics simulations predict enhanced flexibility of R1098Q spectrin with reduced calmodulin (CaM) binding and enhanced susceptibility to calpain cleavage. (**a**) Schematic of intermediate protein complexes simulated with molecular dynamics of spectrin repeats 9–10 with and without bound CaM. (Also see supplemental movie M2). (**b**) Convex bending of the spectrin scaffold (angle between adjacent monomers) is substantially shifted in R1098Q mutants, sampling both a more convex angle than WT spectrin and a broader distribution of values. (**c**) Hydrogen-bonding between D922 and either R1098, Q1098, or K1098 (a hypothetical variant) is markedly different between WT and variant spectrins. Salt-bridging between D922 and R1098 is strongest due to the presence of opposing charges and two hydrogen bond donors. Mutations that conserve native charge (*e.g.* R1098K) could still maintain intermediate hydrogen-bonding and scaffold flexibility, while pathological mutations such as R1098Q fully abrogate hydrogen-bonding and enhance spectrin flexibility. (**d**) The free energy of binding between CaM and spectrin is reduced by 88 kJ/mol in the R1098Q mutant, equivalent to a predicted 15% loss in binding affinity compared to WT complexes.

To better understand how a single residue change could induce such dramatic neuropathology and accelerated spectrin proteolysis, we modeled the impact of the R1098Q substitution using all-atom molecular dynamics simulations of αII spectrin repeats 9 and 10 (Fig. 8a). The in-silico model was constructed by combining a two-repeat crystal structure of chicken αII spectrin (PDB: 1U5P)^33^ and the structure of the human calmodulin-spectrin complex (PDB: 2FOT)^34^. The final system consisted of mouse αII spectrin repeats 9–10 with or without bound CaM in a fully explicit solvent. For comparison, the predicted impact of another analogous mutation, but with charge conservation (R1098K) was also evaluated in silico. These simulations, all of which quickly converged to a stable predicted structure, revealed that R1098 is not in the hydrophobic core of the alpha helix. Thus, the R1098Q mutation does not yield steric clashes. Unexpectedly, the model showed R1098 to be located directly at the junction between spectrin subunits, contiguous with residues linking the 9–10 repeats, and adjacent to the “A/B” loop of the ninth repeat with which it forms a singular salt-bridge. This bridge appears to stabilize the relative orientation of the two repeat units (Fig. 8c).

Substitution of Q or K at R1098 is not likely to substantively modify protein secondary structure, a premise corroborated by both the models and by circular dichroism (CD) measurements of the GST-peptide (supplemental Fig. S4). However, simulations of Q1098 spectrin reveal a striking increase in scaffold bending and flexibility and an increase in the mean inter-repeat angle relative to WT structures (Figs. 8b, supplemental Fig. S5). The Q1098 spectrin two-repeat structure is substantially more convex and flexible, with substantial unmasking of spectrin’s calpain cleavage site even in the absence of CaM. Interestingly, the binding of CaM to the Q1098 variant somewhat attenuates the unmasking of the protease targeted sites. This is the opposite of its effect in the WT protein^14^. CaM also partially mitigates the predicted compaction of the mutated spectrin subunit (Figs. 8b). There is no predicted change in the secondary structure of bound CaM in the Q1098 variant relative to WT spectrin.

To better understand how spectrin flexibility is biophysically modulated, we extracted hydrogen bonding statistics from WT, Q1098, and K1098 variants (Fig. 8c). The changes in hydrogen bonding between protein variants is significant. The WT R1098 residue shares two hydrogen bonds with the nearby D922 residue on the disordered A/B loop, thereby forming a strong salt-bridge that stabilizes scaffold bending. Mutation of this residue to the pathological variant (Q1098) completely abolishes the salt-bridge, resulting in dynamic flexibility. Mutations that partially conserve hydrogen bonding (*e.g.* K1098) are expected to mitigate changes to flexibility, but even the K for R substitution still exhibits reduced h-bond strength with a slightly convex scaffold. In this regard, it is interesting to note that a presumably “conservative” K for R substitution at this site does not appear in evolution in any species examined (Fig. 1). Finally, additional free-energy simulations reveal thermodynamic changes impacting CaM binding to the Q1098 spectrin, resulting in a predicted 15% reduction in affinity (Fig. 8d). This compares favorably to the ≈19% reduction measured experimentally (Fig. 7), and corroborates the hypothesis that this pathological spectrin mutant experiences reduced CaM affinity. The totality of these data indicates that the salt bridge-breaking R1098Q spectrin variant confers substantially more flexibility to the spectrin scaffold, resulting in modified CaM binding and the unmasking of spectrin’s primary calpain-sensitive site (supplemental movie M2). This unmasking is likely to account for the enhanced intrinsic susceptibility of the mutant spectrin to calpain-mediated attack.

## Discussion

Progressive neurodegenerative disorders share many pathologic features yet the identification of common mechanisms of cell injury remains elusive. Data revealed here and in other reports strongly implicate damage to the spectrin scaffold as a feature of both experimental models of neuronal injury^9^ and an event common in Alzheimer’s disease^35–37^, Parkinson’s disease^38^, and several ataxias^24^. Our studies on the R1098Q variant C57BL/6J mouse advance understanding of spectrin’s role in two important respects. While the R1098Q substitution only subtly alters spectrin’s calmodulin affinity it substantially enhances spectrin’s in vivo digestion by calpain, with consequences in the heterozygous animal that are more profound than even total loss of an αII spectrin allele^23^. This suggests that normal regulatory and developmental signals when impinging on a spectrin with an amplified intrinsic response to physiologic calcium/calpain signaling will invoke significant down-stream neuropathology. Conversely, these results also suggest that in conditions characterized by excess Ca^2+^/calpain signaling, as occurs in glutamate or aβ toxicity^4,9^ or following traumatic injury, excessive proteolysis of wild-type spectrin will lead to similar neuropathology. Secondly, these results highlight the importance of substrate-level control of calpain-mediated pathways and validate the in vivo significance of αII spectrin’s 9-10th repeat units as a signaling hub where Ca^2+^/CaM (and tyrosine phosphorylation at Y1176) control local remodeling of the MPS. This conjecture has only been previously explored through in vitro and cell culture studies^9,15,16^.

That spectrin is a common target in several different neurodegenerative disorders suggests that its targeting may be mechanistically important, rather than a passive down-stream consequence of injury. This protein contributes to a bewildering array of cellular processes and hereditary pathologies, well beyond those first associated with the erythrocyte where spectrin was discovered by Marchesi and Steers in 1968. The neuronal MPS shares key topologic features with the erythrocyte, *i.e.* actin filaments cross-linked by spectrin αβ tetramers that link either directly or indirectly through adapter proteins to overlying membrane lipids, receptors, and channels. It differs from the archetypical erythrocyte skeleton in its periodic nature and circumferential orientation about axons and dendrites. The MPS establishes specialized domains in glia and in neurons^22,39–41^. It also mediates receptor tyrosine kinase signaling^20^ and the signals that control axonal remodeling and regression^21^.

Beyond its participation in the MPS, spectrin’s non-canonical roles are also likely to be relevant. Spectrin and spectrin homologues, along with adapter proteins similar to those in the erythrocyte, associate with endomembranes of the ER and Golgi and participate in both secretory and endocytic pathways^42–44^. By linking phospholipid vesicles and selected membrane proteins to the motor proteins dynein and kinesin^42,45,46^, spectrin contributes to retrograde and anterograde vesicular transport along axons and dendrites^47,48^. In polarized cells, spectrin acts upstream with actomyosin in the Hippo pathway to modulate YAP/TAZ signaling responses to mechano-stimulation^49,50^.

Collectively, the diversity of biologic processes in which spectrin plays a fundament role provide fertile areas for future investigation of their linkage to endocytosis, autophagy, aβ trafficking, protein sorting, proteostasis, Ca^2+^/CaM/calpain activation and axonal and dendritic integrity. These are all processes that have been associated with various neurodegenerative disorders. The data presented herein suggests that inappropriate regulation of just the neuronal spectrin skeleton alone is sufficient to activate a cascade of events leading to progressive ataxia and neuronal degeneration. These results thus highlight the relevance of understanding in greater depth the molecular mechanisms of neuronal spectrin function as it relates to the diverse pathologies of ataxia and neurodegenerative disease.

## Materials and methods

### Mice

C57BL/6J mice were housed under pathogen-free conditions and maintained as a breeding colony. Experiments involving mice were approved by The Local Ethical Committee in Wroclaw (Poland) under permission number 78/2018 and by The Institutional Animal Care and Use Committee (IACUC) at Yale University, which is accredited by the Association for the Assessment and Accreditation of Laboratory Animal Care (AAALAC). Both genders of mice were used without preference. All methods were performed in accordance with the relevant approved guidelines and regulations.

All animal studies also were conducted in compliance with the ARRIVE guidelines (PLoS Bio 8(6), e1000412,2010) (https://www.nature.com/srep/journal-policies/editorial-policies#experimental-subjects). Addressing the ARRIVE Essential ten specifically: (1) Study design – Compared heterozygous versus WT SPTAN1 R1098Q mice; study unit was single animals. (2) Sample size – Based on available animals of a given age and sex, no a priori sample size calculation performed. For age (wks): 4(n = 5); 10(n = 12); 24(n = 14); 54(n = 12). Total mice tested for coordination = 43; (3) Inclusion/exclusion criteria – Inclusion criteria were age, sex, and genotype. Sex balanced groups were preferred where possible. A priori exclusion criteria were any mice that weighed less than 20% of the normal for age at the time of experiment. There turned out to be no exclusions in any experimental group. (4) Randomization – No randomization was used to allocate mice to groups. The mice in every experimental group were measured in random order by two independent observers. Cage location was changed every week. (5) Blinding – J.S. and M.Z. allocated mice and conducted the experiments; J.S. and A.M. assessed the outcome and analyzed the data. (6) Outcome measures – Rotarod and parallel rod performance. (7) Statistical methods – One or two-way ANOVA to assess interactions between genotype, age, and performance. Bonferoni post tests. *p* values > 0.05 considered non-significant; exact p values presented throughout manuscript. (8) Experimental animals—Laboratory mice (Mus musculus), genetic background strain C57BL/6J  C57Bl/6J, point mutation in Spna-2 (SPTAN1) gene R1098Q; males and females, 4–54 wks old. Genetically altered mice (spontaneous mutation) were identified at Hirszfeld Institute of Immunology and Experimental Therapy, PAS, Wroclaw, Poland. For backcross C57BL/6J C57Bl/6 mice from the Center of Oncology, Warsaw, Poland were used. Sentinel animals were tested every 3 months for the absence of parasites, bacterial infections. All mice were immune competent. (9) Experimental procedures – Testing protocols were deemed appropriate based on established literature (see relevant methods sections). Mice were acclimatized to the rotarod and parallel rod tests for 3 days before testing. Every mouse was given three trials during testing. (10) Results – Summarized with statistics in supplemental Fig. S1.

### Sequencing

Whole exome sequencing was conducted on two male, littermate mice; one without and one with the ataxic phenotype. Standard techniques were used to conduct whole exome sequencing including exome capture, mapping reads and mutational analysis. Exome capture was performed and mapping reads (Illumina HiSeq bcl files) were processed using the BCLConvertor. All reads were formatted into fastq files and aligned to mouse reference genome GRCm38/mm10 (NCBI) using BWA (bwa-0.5.9-R16) with default parameters. BAM files generated from alignment and mutation detection using Ensembl variant predictor led to the identification of a non-synonymous missense variant for Sptan1. The validity of this substitution was verified by Sanger sequencing. A full list of the 34 non-synonymous mutations identified by WES of the littermates is tabulated in Supplemental Table S1.

To follow the mutant Sptan1allele through multiple generations, mice were analyzed by PCR amplification from genomic DNA of a 256 bp region that encompassed the c.3293G > A variant. Digestion of the resulting amplimer with *MspI* endonuclease allowed distinction of the WT from variant allele by agarose gel electrophoresis. The primers utilized for the PCR reaction were: forward primer 5′-ctgccttccttgtccattgt-3′; reverse primer 5′-agacagcccataccttctgg-3'.

### Coordination

Rotarod testing followed published procedures^51^ on a group mice between aged between postnatal day 26 up to mice that were 386 days old. Approximately equal numbers in each group were wild-type or heterozygous R1098Q mice. Each mouse was tested three times. In a second evaluation, gait analysis of was performed in a confined walkway 10 cm wide by 30 cm long^51,52^. After dipping their paws into ink, 6–8 week old mice freely walked down the corridor on white paper. The distance of each step was determined by measuring the distance between the hind limb paw prints. In a third analysis, 3–4 week or 54 week old animals were placed on a platform consisting of parallel rods, and the number of times the animals failed to reliably place their feet on the rods was tabulated. This test is considered a measure of both ataxia and locomotion^53^.

### Microscopy

For light microscopy, brains were fixed in 4% paraformaldehyde (PFA) overnight, embedded in paraffin and sectioned at five microns. Slide preparation was done by the Yale Pathology Tissue Services core facility at Yale University (https://medicine.yale.edu/pathology/ypts/). Slides were cleared in xylene, rehydrated in graded aqueous ethanol and distilled water and warmed to 95 °C in 0.1 M Na-citrate buffer at PH 6.0. Slides were then placed in Tris-buffered saline (TBS) (50 mM Tris, 150 mM NaCl, pH7.5) with 0.1% Tween (TBS-T). Hydrogen peroxide (3% final concentration) was added to quench endogenous peroxidase activity. The primary antibody diluted in TBS/T was applied for 1 h followed by TBS-T washes and a 45 min incubation with horseradish peroxidase conjugated secondary antibody (Jackson ImmunoResearch). Diamino Benzidine (DAB) solution is then applied for 5 min to brain sections to generate a reaction product. Sections were counterstained in hematoxylin, and visualized and imaged using an Orca digital camera with SPOT Basic 5.6 imaging sofware (SPOTIMAGING, Sterling Heights, MI; http://www.spotimaging.com/software/spot-basic/).

For immunohistochemistry (IHC), all commercial antibodies were diluted in TBS-T at 1:100 and the two laboratory prepared antibodies (anti-βIII and anti-BDP1) were diluted in TBS-T at 1:500. The laboratory prepared anti-spectrin antibodies were affinity purified rabbit polyclonal anti-βIII spectrin^54^ and an antibody that is specific to the calpain-cleaved fragment of αII spectrin (anti-BDP1)^9^. Commercially available monoclonal antibodies used in this study were anti-αII spectrin, clone D8B7, RRID:AB_2564660 (Biolegend); anti-β-Actin, clone AC-74, RRID:AB_476697 (Sigma); and anti-GFAP, clone G-A-5, RRID:AB_2314539 (Sigma). Commercial rabbit polyclonal antibodies used were anti-EAAT4, RRID:AB_1622384 (Alpha Diagnostics); and anti-calbindin, RRID:AB_213554 (Chemicon).

For electron microscopy, brain sections were fixed in 4% PFA, then post-fixed 2 h at 4 °C in 2% glutaraldehyde, 0.1% tannic acid in 0.1 M Na cacodylate, pH 7.4; permeated 1 h in 1% OsO4, pH 7.6; dehydrated through graded ethanol (40, 60,70, 80, and 96%); then embedded in a 50/50 mixture of acetone and epon (Embed-812). Ultrathin sections (80 nm) were stained with 2% uranyl acetate and lead citrate. Grids were examined and imaged on a Zeiss electron microscope housed in Yale Department of Cellular and Molecular Physiology Imaging Core.

### Blotting

Dissected cerebella were immediately frozen in liquid nitrogen, lysed in ice cold lysis buffer (50 mM pH 7.4 Tris–HCl, 1% Triton X-100, 0.25% sodium deoxycholate, 150 mM NaCl, 1 mM EDTA) supplemented with protease inhibitors (Complete Protease Inhibitor Cocktail Tablets provided in EASYpack, Cat No. 04 693 116 001, Roche, Germany) in the BeadBug™ Microtube Homogenizer Model D1030-E, Benchmark Scientific, USA (4,000 rpm for 45 s, 1.5 mm zirconium beads). Insoluble material was removed by centrifugation for 20 min at 15,000 rpm. Equal volume of Laemmli buffer containing β-mercaptoethanol was added to supernatants and heated for 5 min at 95 °C then evaluated by SDS-PAGE. For Western analysis anti-αII spectrin, clone AA6 (at 1:500), RRID:AB_11214057 (Chemicon), βIII spectrin, clone 3B1NB (1:850) and β-Actin, clone AC-74 (1:5000), RRID:AB_476697 were used.

### Binding

Surface plasmon resonance (SPR) was used to measure the binding of purified bovine brain calmodulin (CaM) (P1431, Sigma-Aldrich) to recombinant GST-αII spectrin peptides representing spectrin codons 891-1231. This 341 residue sequence represents repeats 9 and 10 along with the inserted calpain and calmodulin interaction sites. PCR was used to generate from cDNA the genomic sequence encoding these repeats. αII spectrin amplimers were subcloned into pGEX-6P-1 using BamHI and NotI restriction sites. For fusion protein production, CodonPlus-RIL. E. coli were transformed with plasmid vectors encoding WT or mutant αII spectrin peptides. Recombinant peptides were purified by GST-affinity column chromatography. Their binding to CaM was analysed by SPR using a BIAcore T200 instrument (GE Healthcare). The CM5 sensor chip surface was activated for direct amine coupling of the goat anti-GST antibody (αGST-Ab, #27457701 (GE Healthcare)) by injecting 40 μl of degassed coupling solution (HBS-NHS/EDC) (0.01 M HEPES, pH 7.4, 0.15 M NaCl, 0.05 M NHS (N-hydroxysuccinimide), 0.2 M EDC (N-ethyl-N’-dimethylaminopropyl carbodiimide) followed by ethanolamine injection after the αGST-Ab was bound so as to deactivate esters on the sensor chip surface. Purified WT or R1098Q αII spectrin peptides were then bound to the prepared surface via their GST-tag to a final level of 1,000 response units (RU). Increasing concentrations of CaM (20 nM to 500 nM) in HBS-NHS running buffer with 2 mM CaCl2 were passed over the ligand-immobilized chip surface (association phase), followed by dissociation with running buffer. The same samples were passed over a control chip surface with only the anti-GST antibody immobilized. All binding experiments were carried out at 25 °C at a 20 µl minute^−1^ flow rate. Sensorgrams were obtained by subtracting the buffer blank from the recorded curves. Then, the curves recorded when calmodulin was passed over the blank sensor surface were subtracted. The equilibrium constants (K_D_), defined as a k_d_/k_a_ ratio, were determined using BIAevaluation 3.1 software available at https://www.biacore.com/lifesciences/service/downloads/software_licenses/biaevaluation/. For global fitting, a 1:1 Langmuir binding model with an included mass transport step was applied based on criteria provided by the BIAevaluation handbook https://www.biacore.com/lifesciences/service/downloads/Handbooks. Residuals were evaluated for systematic divergences from the fitting algorithms as a measure of the appropriateness of the binding model.

### Modeling

#### Molecular structures

In order to evaluate structural differences between WT and R1098Q variants of αII spectrin in mice, a homologous two-unit chicken protein (repeats 15 and 16) was utilized^55^ as an initial scaffold (PDB: 1U5P). Subsequently, each amino acid was renumbered and changed to those in mouse repeats 9 and 10 using the BioLuminate package (Schrödinger, LLC), followed by N-terminal acetylation and C-terminal methylation to neutralize charged ends. The alternative transcript and the SH3 domain inserts were not included in the final structure. A prior CaM structure bound to its site in the 10^th^ repeat of spectrin^34^ (PDB: 2FOT) was loaded into BioLuminate, where helical residues were mutated to match its corresponding site in repeat 10. Similarly, amino acids missing in the crystal structure of CaM were added, and the entire complex was attached to helix C of spectrin using random coil amino acids. The resulting structure (repeats 9 and 10 of mouse αII spectrin with the disordered calpain and CaM binding site complexed to CaM) was energy-minimized with BioLuminate until the structure no longer changed conformation. From the initial system, six subsystems were constructed representing chicken WT, mouse WT, mouse WT without CaM, mouse R1098Q mutant, mouse R1098Q mutant without CaM, and a putative R1098K mutant (with and without bound CaM) as a control. In addition to bound Ca^2+^, which stabilizes CaM, potassium ions were added to neutralize the net charge of spectrin in each subsystem (33/32 K^+^ for WT/ mutant spectrin, respectively).

#### Molecular modeling

Atomistic molecular dynamics simulations were carried out on each structure using the GROMACS 2020 integrator^56,57^. Each system utilized the CHARMM36m force field for proteins^58,59^ and was subsequently solvated with ~ 100,000 explicit TIP3P water molecules^60,61^. This resulted in a simulation box that was approximately 13 × 15 × 15 nm^3^ and contained about 280,000–300,000 atoms. Newton’s equations of motion were integrated using a 2 fs time step under a leap frog algorithm. Short-range van der Waals and Coulomb potentials were truncated at 1.2 nm, while long-range electrostatics were tabulated with Particle Mesh Ewald summation^62^, which reduces computation through Fast Fourier Transforms. Cartesian periodic boundary conditions were also implemented in all directions to minimize the effects from unit-cell boundaries. In each system, an NPT ensemble (constant number of atoms, pressure, and temperature) was maintained at 300^o^K and 1 bar of isotropic pressure using a weakly-coupled Berendsen barostat and thermostat^63–65^. Protein chemical bonds containing hydrogen atoms were rigidly constrained using the linear constraint solver (LINCS)^66^, while water bonds were constrained using the SETTLE algorithm^67^ that utilizes Lagrange multipliers to maintain holonomic constraints under a symplectic integrator. Each simulation was, once again, energy-minimized in GROMACS for 5000 steps using a force tolerance of 750 kJ mol^-1^ nm^-1^. Following this, each system was simulated for at least 400 ns. To confirm the convergence of data during this time, an additional 200 ns simulation was carried out afterwards (for a total of 600 ns.) No major conformational changes were observed after 400 ns. Similarly, the first 100 ns of each simulation was ignored during analysis to ensure that each system reached equilibrium. Simulations were carried out on either the Grace supercomputing cluster, maintained by the Yale Center for Research Computing (YCRC), or the Stampede2 cluster at the Texas Advanced Computing Center (TACC) through the XSEDE program.

#### Potentials of mean force measurements

Following molecular dynamics simulations, additional enhanced-sampling simulations were carried out to deduce the binding affinities between CaM and spectrin in WT and the R1098Q variant spectrin using umbrella sampling^68^. CaM was initially pulled at a rate of 3E-4 nm/ps away from the spectrin scaffold, using a virtual spring (K = 5000 kJ mol^-1^ nm^-2^). About 50 umbrella windows were sampled in 0.1 nm bins, traversing a total of 5 nm. Each umbrella replicate was run for 20 ns, totaling 1 µs, using a Nosé-Hoover thermostat^69^ under a constant volume (NVT ensemble). Following this, a weighted histogram analysis method (WHAM)^70^ was used to reweight each replicate, yielding a free energy of binding (ΔG) between CaM and αII spectrin. Statistical errors were estimated using bootstrapping techniques^71^ where approximately 200 bootstraps informed the uncertainty of the binding affinity.

#### Molecular dynamics analysis

Simulation images and movies were rendered using VMD 1.9.1^72^. C_α_-RMSD measurements were extracted from each system using the GROMACS tool ‘gmx rms’, while secondary structures were tabulated using both the ‘gmx dssp’ tool^73^ and custom Perl scripts. Protein bending and compression was measured using the analysis tools ‘gmx angle’ and ‘gmx distance’ that was constructed from C_α_ atoms residing on V960, R1003, and A1026 residues. These represent central and distal positions on the spectrin triple-helix backbone. Additionally, intra- and intermolecular hydrogen bonds were deduced using the GROMACS tool ‘gmx hbond’.

### Analysis

Cell counting data was collected in triplicate by three independent and “blinded” observers. Experimenters were blinded in all experiments except for the movement studies due to the severity of the phenotype, which was immediately obvious to any observer. Data was analyzed using Microsoft Excel and GraphPad Prism. All error bars are ± SEM unless otherwise indicated. Unpaired, one or two-tailed (as indicated) Student’s t-test as implemented in Excel version 14.7.3 for the Mac 2011 was used for comparisons between WT and R10989Q values. Alternatively, where indicated Two-tailed or one-tailed ANOVA calculations as appropriate were used to determine significance. Exact p values are presented in the text or legends. Images were formatted for presentation in Adobe Photoshop ver. 12.1. The only image processing used was “autocontrast” applied once to all microscopic images used for presentation (but not for quantitative measurements, in which images were used unprocessed). For the quantitation of GFAP in the cerebellum, the overall GFAP intensity in multiple respective sections was measured as the overall intensity in the inverted blue channel, since this was found to yield the greatest discrimination between the immunoperoxidase stained GFAP positive astrocytes and the counterstained background.

## Supplementary Information

Supplementary Legends.

Supplementary Information.

Supplementary Video 1.

Supplementary Video 2.

## Data Availability

The data that support the findings of this study are available from the corresponding authors upon reasonable request. The WES data from the WT and R1098Q littermates is available as BioProject ID PRJNA682493 (https://www.ncbi.nlm.nih.gov/sra/PRJNA682493).

## Code availability

Custom Perl scripts used in the modeling studies are available upon request.
