# Publisher Correction: Age-dependent ataxia and neurodegeneration caused by an αII spectrin mutation with impaired regulation of its calpain sensitivity

**DOI:** 10.1038/s41598-021-97068-y

**Published:** 2021-09-08

**Authors:** Arkadiusz Miazek, Michał Zalas, Joanna Skrzymowska, Bryan A. Bogin, Krzysztof Grzymajło, Tomasz M. Goszczynski, Zachary A. Levine, Jon S. Morrow, Michael C. Stankewich

**Affiliations:** 1 Department of Tumor Immunology, Hirszfeld Institute of Immunology and Experimental Therapy, Polish Academy of Sciences, Weigla 12, 53‑114 Wrocław, Poland; 2 Department of Molecular Biophysics and Biochemistry, Yale University, New Haven, CT USA; 3 Department of Biochemistry and Molecular Biology, Wroclaw University of Environmental and Life Sciences, Norwida 31, 50‑375 Wrocław, Poland; 4 Department of Pathology, Yale University School of Medicine, 310 Cedar Street, LH108, New Haven, CT 06520 USA; 5 Department of Molecular, Cellular, and Developmental Biology, Yale University, New Haven, CT USA

Correction to: *Scientifc Reports* (2021) https://doi.org/10.1038/s41598-021-86470-1, published online 31 March 2021

The original version of this Article contained typographical errors in Figure 1b where there was erroneous spacing in the sequence alignments. The original Figure 1 and accompanying legend appear below.

**Figure 1:**
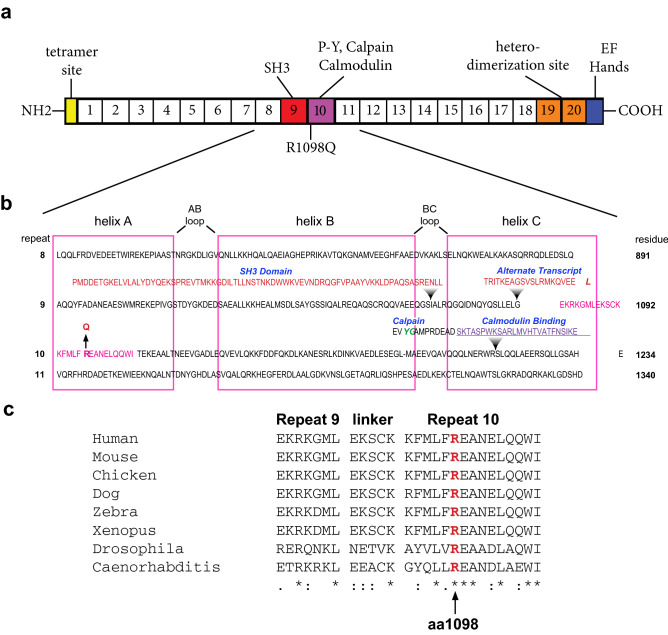
Relationship of the R1098Q variant to αII spectrin structure. (**a**) Schematic representation of αII spectrin with 20 complete homologous repeats each ≈ 106 residues and the location of selected functional domains that interrupt the repeat motif. The 9th and 10th repeats (red and purple respectively) harbor an inserted SH3 domain, a variably appearing alternative transcript insertion, and specialized sequences encompassing the site of CaM binding and the site Y1176 and G1177 that is preferentially cleaved by calpain. (**b**) Sequence of repeats 8–11 aligned by homology. The boxes denote the boundaries of each of the three α-helices (A–C) of the canonical repeat unit; these are separated by AB and BC loops and inter-unit linkers. The sequences representing the SH3 domain and the alternative transcript inserted into repeat 9 and the calpain and CaM interaction sites in repeat 10 are indicated. The arrow marks the site of the R1098Q mutation near the start of the 10^th^ repeat. The YG site of preferred calpain cleavage is colored green. (**c**) Clustal alignment of residues flanking the R1098Q mutation. This locus, and the flanking region, is almost perfectly conserved across diverse species. Residues fully conserved are denoted with asterisks (*); residues strongly conserved with a score greater than 0.5 on the PAM250 matrix are designated with a colon (:). Weakly similar residues are denoted with a period (.).

The original Article has been corrected.

