# Supplementary material for: Age-dependent ataxia and neurodegeneration caused by an αII spectrin mutation with impaired regulation of its calpain sensitivity
Source: Sci Rep. 2021 Mar 31;11:7312. doi: 10.1038/s41598-021-86470-1 (PMC8012654; doi:10.1038/s41598-021-86470-1)
Supplement: Supplementary file 1 — Supplementary Legends. [file 41598_2021_86470_MOESM1_ESM.docx]

**Supplemental Material**

**Figure S1: Coordination testing of R1098Q mice.**

(**a**) Beginning at 4 weeks of age, mice were subjected to a battery of tests to establish the progressivity of their ataxia. Two rotational speeds of the rotarod were used, 8 and 14 rpm, which were slow enough to allow sensitive detection of the threshold when the performance of heterozygous mice diverged from their WT littermates ^[51](#_ENREF_51)^. When tested at 8 rpm, this occurred after about 20 weeks of age; at 14 rpm, the onset of the phenotype was manifest by 9-10 weeks of age. Thereafter, performance continued to deteriorate. Each data point represents the mean of three tests on a separate mouse. In total, 43 mice were tested (N = 5 at 28 d; 12 at 70 d; 14 at 168 d; and 12 at 378 d). Data analyzed by pair-wise (by age) single-tail ANOVA. (**b**) Gait was evaluated in 6-8 week old mice by allowing them to freely walk down a corridor on white paper after dipping their paws into ink. The distance for each step was determined by measuring the distance of the hind limb paw prints. Only subtle and insignificant differences were observed in stride length and the animals had equal sway. N = 2 WT and 3 het mice. (**c**) Parallel rod open field floor testing revealed that heterozygous (het) 54 week old mice made significantly more missteps per meter than their WT littermates. (Note, testing 3-4 week old mice showed no difference from WT in this test, data not shown). This test is a measure of both ataxia and locomotor activity[^53^](#_ENREF_53). Their performance over four repetitive trials was highly reproducible and significant by pair-wise single tail ANOVA analysis. On average, the R1098Q heterozygotes were 2.7 times more likely to misstep. Each data point represents a separate mouse (N= 5 WT and 5 het mice).

**Figure S2:**  **In-utero comparison of E18 fetal mice.** Fetus on left genotypes as WT αII spectrin. The two fetuses on the right genotype as homozygous for the mutant αII spectrin with Q at residue 1098 instead of R. Homozygotes die by E18 with severe craniofacial, neurodevelopmental, and vascular defects.

**Figure S3: Cerebellum of P7 mice. ** Cerebellum from postnatal day 7 mice immunostained for βIII spectrin. There is little if any detectable difference between WT and R1098Q heterozygotes in Purkinje cell morphology at this age. Also see Fig. 2.

**Figure S4: Secondary structure predictions. (a)** Secondary structure predictions derived from molecular dynamics simulations indicate that no major structural changes occur as a result of spectrin mutations or calmodulin (CaM) binding. Systems include both wild type (WT) and mutant (R1098Q) spectrin variants modeled across repeats 9-10, primarily recapitulating the native A, B, and C helical segments. **(b)** Experimental CD spectra of the WT or mutant spectrin GST-9-10 repeat peptide. Calculated secondary structure is as listed in table. The measured structure closely approximates that derived from in-silco modeling.

**Figure S5: Compaction of the repeat unit.**

Distribution of spectrin repeat unit lengths from molecular dynamics indicate that the repeat unit with CaM binding activity (red) tends to be shorter than the preceding spectrin repeat (blue). The R1098Q spectrin lengths (mutant) are also shorter and more variable than their WT counterparts. In some instances, bi-modal lengths were also observed, indicating the presence of small oscillations in spectrin length.

**Figure S6: Western blots.**

The gels used for quantification and shown in Fig. 3 were truncated and cropped for clarity. The full-length gels are presented here.

**Figure S7: Quantifying changes in cerebellum**

1. Density of PC’s along the PC layer, comparing WT with R1098Q heterozygous (het) mice at two ages. While there is no difference in P7 (1 week old) mice, most PC’s are lost within 6 months. For each of the four categories (WT or het at two ages), three independent fields of view (FOV) were evaluated for each of two mice (as represented by the symbol shapes, dots and triangles) for a total of 24 fields over 8 mice). All data points are shown. The significance of variation in the counts per FOV was evaluated by single-tail T-test.
2. Molecular layer width in 26 week and 1.5 year old mice. By 26 weeks, there is substantial thinning of the molecular layer. The change at 1.5 years is similar. Layer widths were evaluated in four to six independent FOVs for each of two mice at 26 weeks, and in six FOVs for the single mouse available at 1.5 yrs. The significance of variation in the widths per FOV was evaluated by single-tailed T-test.
3. GFAP stain intensity in cerebellum. The mean immune-positive stain intensity of GFAP was determined by averaging over independent FOVs in the inverted blue channel, which best discriminates GFAP immunoperoxidase staining from background counterstaining. Note the enhanced GFAP levels in hets versus WT animals, and that the average GFAP intensity increased with age in both. Three to six FOVs were evaluated in each group. All data points are shown. Two animals were available in each group at 26 weeks; only a single animal was available in each group at 1.5 ys. Each data point represents a separate measurement on separate animals; separate animals are denoted by symbol shapes (dots or triangles). Data analyzed by single tail, 2-sample homosedastic T-test.

**Additional Supplemental Materials - Movies**

**Movie M1**: Ataxic phenotype of nine month old C57BL/6J mouse carrying the R1098Q mutation versus WT littermate. The mutant mice exhibits obvious motor and coordination difficulty, which is fully developed by this age.

**Movie M2**: Molecular models of WT (R1098) spectrin (beginning of movie) versus Q1098 spectrin (end of movie) demonstrating the enhanced exposure of the calpain cleavage site at Y1176 (colored blue) in the Q1098 spectrin. This increased exposure at Y1176 is postulated to up-regulate spectrin’s steady-state calpain interactions.

**Table S1: Variants (non-synonymous) identified by WES in ataxic C57B/6J mice**
