# Supplementary material for: Age-dependent ataxia and neurodegeneration caused by an αII spectrin mutation with impaired regulation of its calpain sensitivity
Source: Sci Rep. 2021 Mar 31;11:7312. doi: 10.1038/s41598-021-86470-1 (PMC8012654; doi:10.1038/s41598-021-86470-1)
Supplement: Supplementary file 2 — Supplementary Information. [file 41598_2021_86470_MOESM2_ESM.pdf]

Supplemental Material

Age-dependent ataxia and neurodegeneration caused by an αII spectrin mutation with impaired regulation of its calpain sensitivity

Arkadiusz Miazek[1,3], Michał Zalas[1], Joanna Skrzymowska[1], Bryan A. Bogin[2], Krzysztof Grzymajło[3], Tomasz Goszczynski[1], Zachary A. Levine[2,4], Jon S. Morrow[4,5,^] and Michael C. Stankewich[4,^]

1 Department of Tumor Immunology, Hirszfeld Institute of Immunology and Experimental Therapy, Polish Academy of Sciences, Weigla 12, 53-114 Wroclaw, Poland
2 Department of Molecular Biophysics & Biochemistry, Yale University, New Haven, CT, U.S.A
3 Department of Biochemistry and Molecular Biology, Wroclaw University of Environmental and Life Sciences, Norwida 31, 50-375 Wroclaw, Poland
4 Department of Pathology, Yale School of Medicine, New Haven, CT, U.S.A
5 Department of Molecular, Cellular, and Developmental Biology, Yale University, New Haven, CT, U.S.A
^ Co-senior Authors

Supplemental Figure Legends

Figure S1: Coordination testing of R1098 and Q1098 mice.
Figure S2: In-utero comparison of fetal mice.
Figure S3: Comparison of WT vs. heterozygote cerebellum immunostained for βIII-spectrin at postnatal day 7.
Figure S4: Secondary structure predictions
Figure S5: Compaction of the spectrin repeat unit relaxed in mutant
Figure S6: Original un-cropped Western blots used in Figure 3.
Figure S7: Measured cerebellar changes with age.
Table T1: Variants identified by WES

Movie M1: WT vs. R1098Q mice at 9 months of age.
Movie M2: Dynamics simulation of WT vs Q1098 spectrin ± calmodulin; Y1176 (calpain cleavage site) is colored blue.

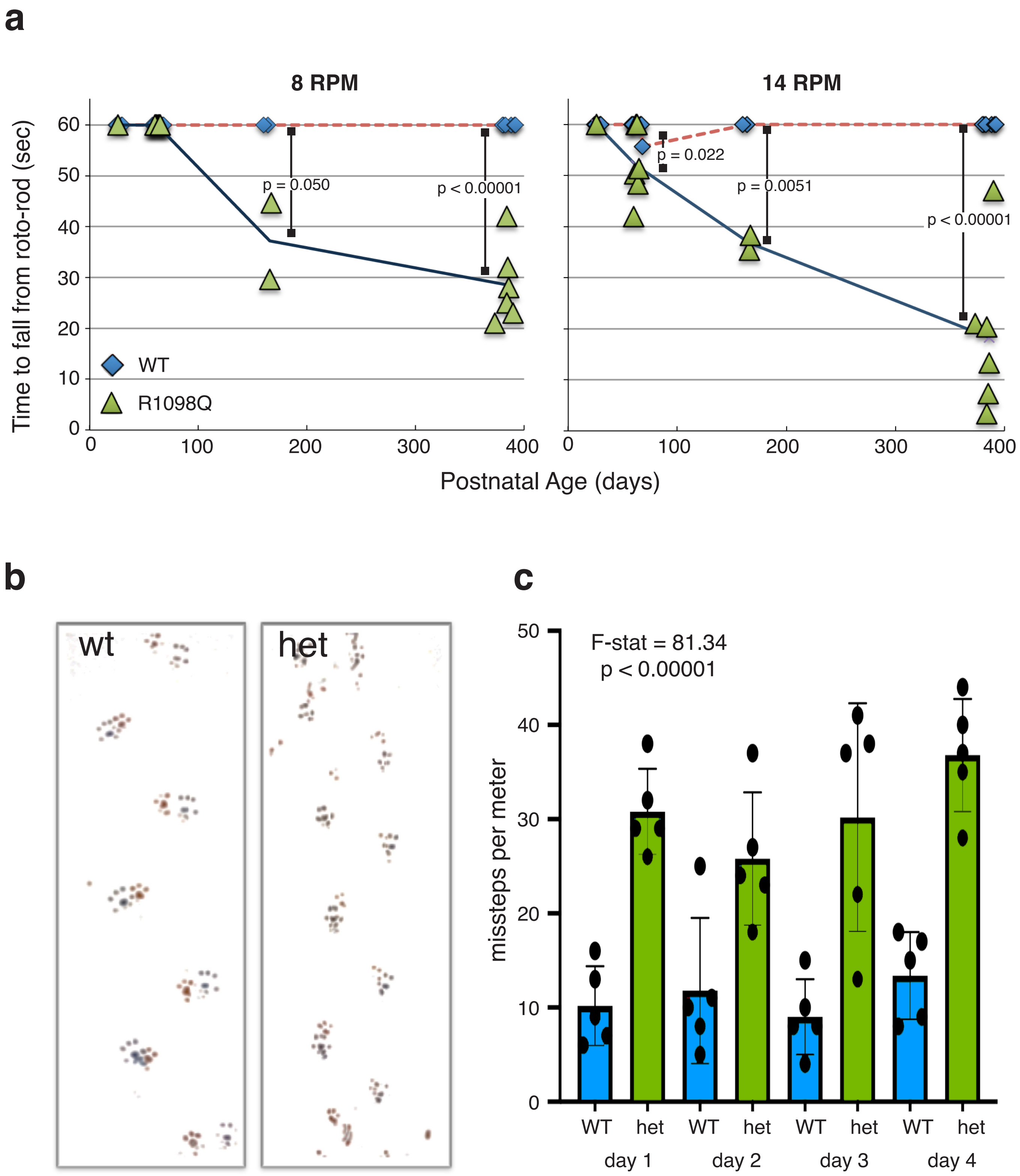

Supplemental Figure S1: **- Coordination testing of R1098Q mice**

Age-dependent ataxia and neurodegeneration caused by an αII spectrin mutation with impaired regulation of its calpain sensitivity

Arkadiusz Miazek, Michał Zalas, Joanna Skrzymowska, Bryan A. Bogin, Krzysztof Grzymajło, Tomasz Goszczynski, Zachary A. Levine, Jon S. Morrow, and Michael C. Stankewich

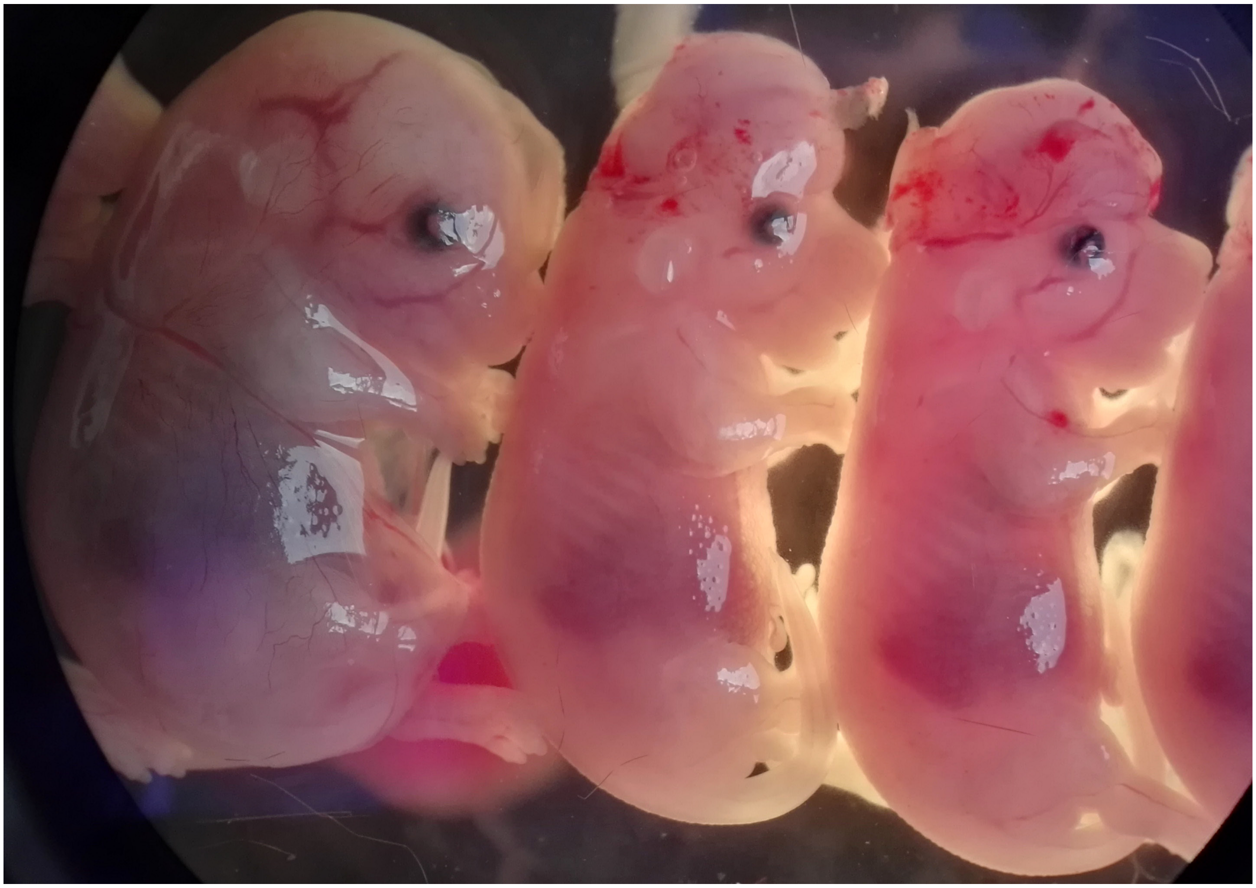

Wild Type
αII spectrin

R1098Q
αII spectrin
homozyous

R1098Q
αII spectrin
homozyous

Supplemental Figure S2 - In-utero comparison of fetal mice

Age-dependent ataxia and neurodegeneration caused by an αII spectrin mutation with impaired regulation of its calpain sensitivity

Arkadiusz Miazek, Michał Zalas, Joanna Skrzymowska, Bryan A. Bogin, Krzysztof Grzymajło, Tomasz Goszczynski, Zachary A. Levine, Jon S. Morrow, and Michael C. Stankewich

P7 brain stained with anti-βIII spectrin

WT

R1098Q het

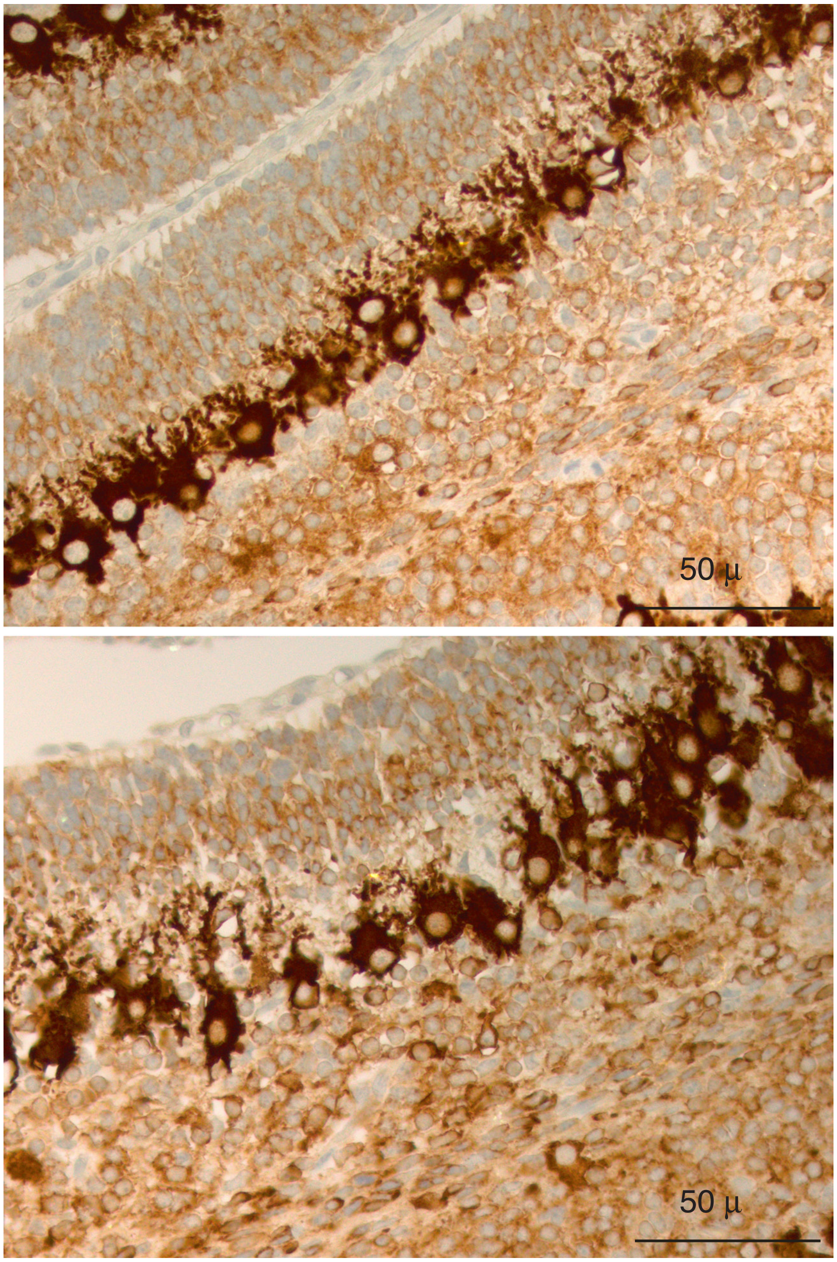

Supplemental Figure 3: Comparison of wt vs heterozygote cerebellum immunostained for βIII-spectrin at postnatal day 7.

Age-dependent ataxia and neurodegeneration caused by an αII spectrin mutation with impaired regulation of its calpain sensitivity

Arkadiusz Miazek, Michał Zalas, Joanna Skrzymowska, Bryan A. Bogin, Krzysztof Grzymajło, Tomasz Goszczynski, Zachary A. Levine, Jon S. Morrow, and Michael C. Stankewich

a

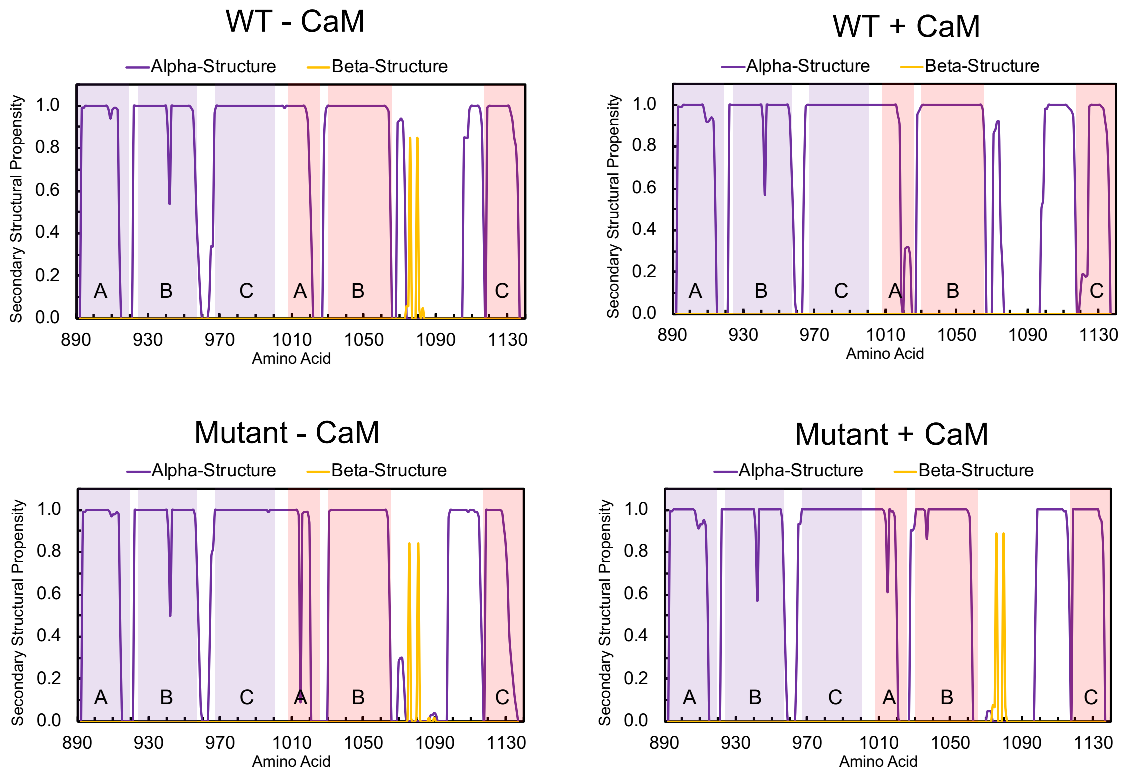

b

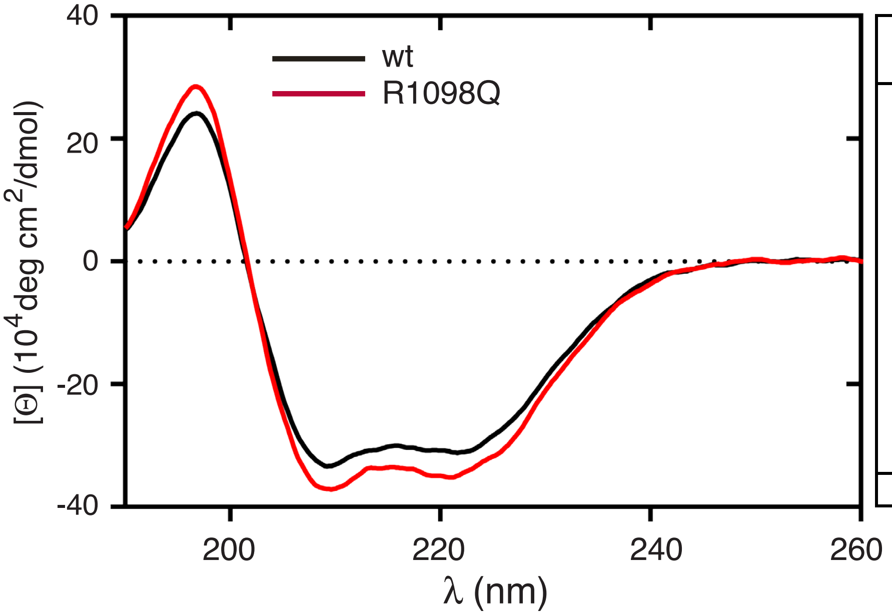

Structure	Wild Type	R1098Q
helix1	0.59	0.57
helix2	0.17	0.19
strand1	0.05	0.04
strand2	0.01	0.02
turns	0.09	0.08
unordered	0.09	0.08
Total	1.00	0.98

Supplemental Figure S4 - Secondary Strucure Predictions.

Age-dependent ataxia and neurodegeneration caused by an αII spectrin mutation with impaired regulation of its calpain sensitivity

Arkadiusz Miazek, Michał Zalas, Joanna Skrzymowska, Bryan A. Bogin, Krzysztof Grzymajło, Tomasz Goszczynski, Zachary A. Levine, Jon S. Morrow, and Michael C. Stankewich

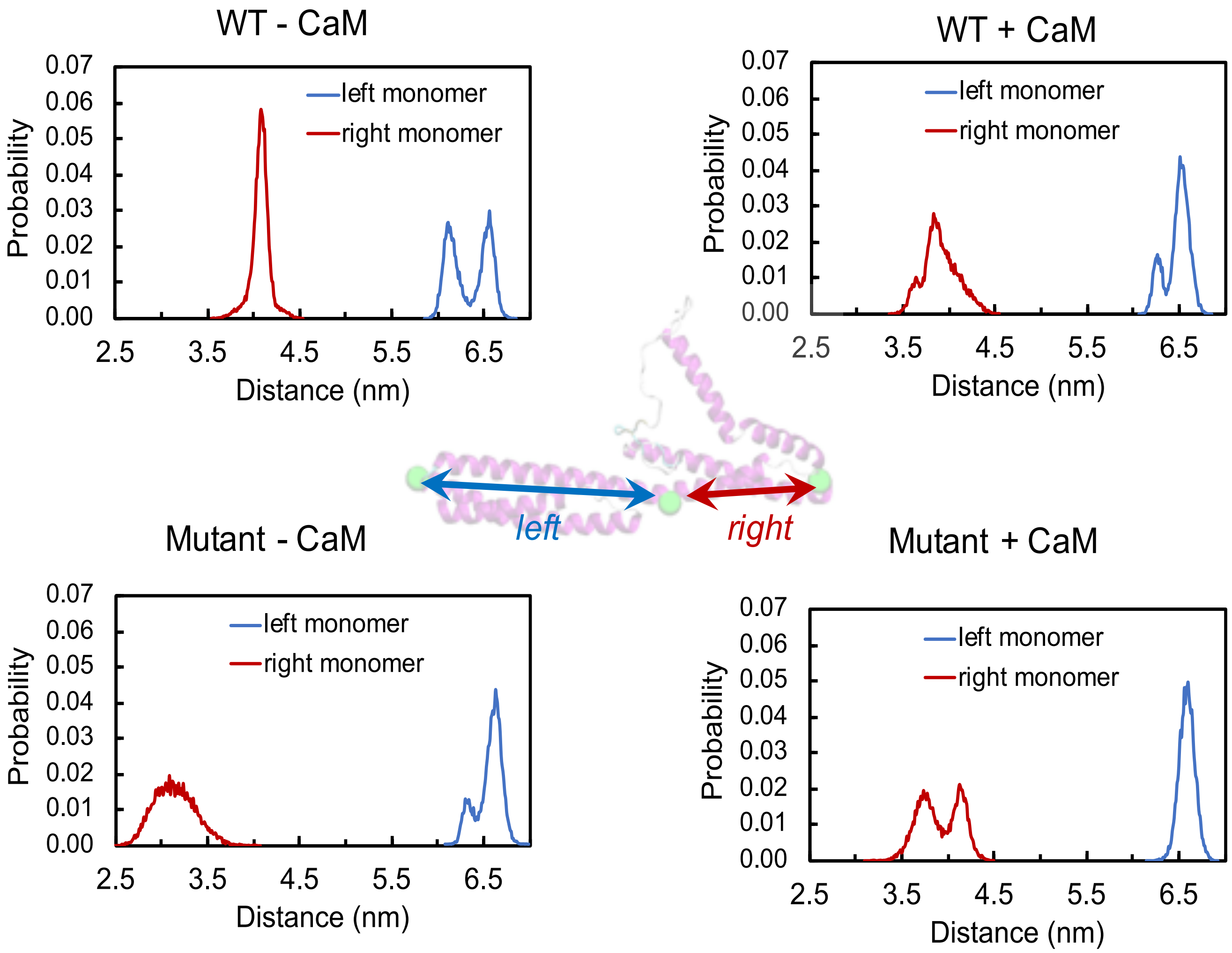

Supplemental Figure S5 - Compaction of the spectrin repeat unit relaxed in mutant

Age-dependent ataxia and neurodegeneration caused by an αII *spectrin mutation with impaired regulation of its calpain sensitivity*

Arkadiusz Miazek, Michał Zalas, Joanna Skrzymowska, Bryan A. Bogin, Krzysztof Grzymajło, Tomasz Goszczynski, Zachary A. Levine, Jon S. Morrow, and Michael C. Stankewich

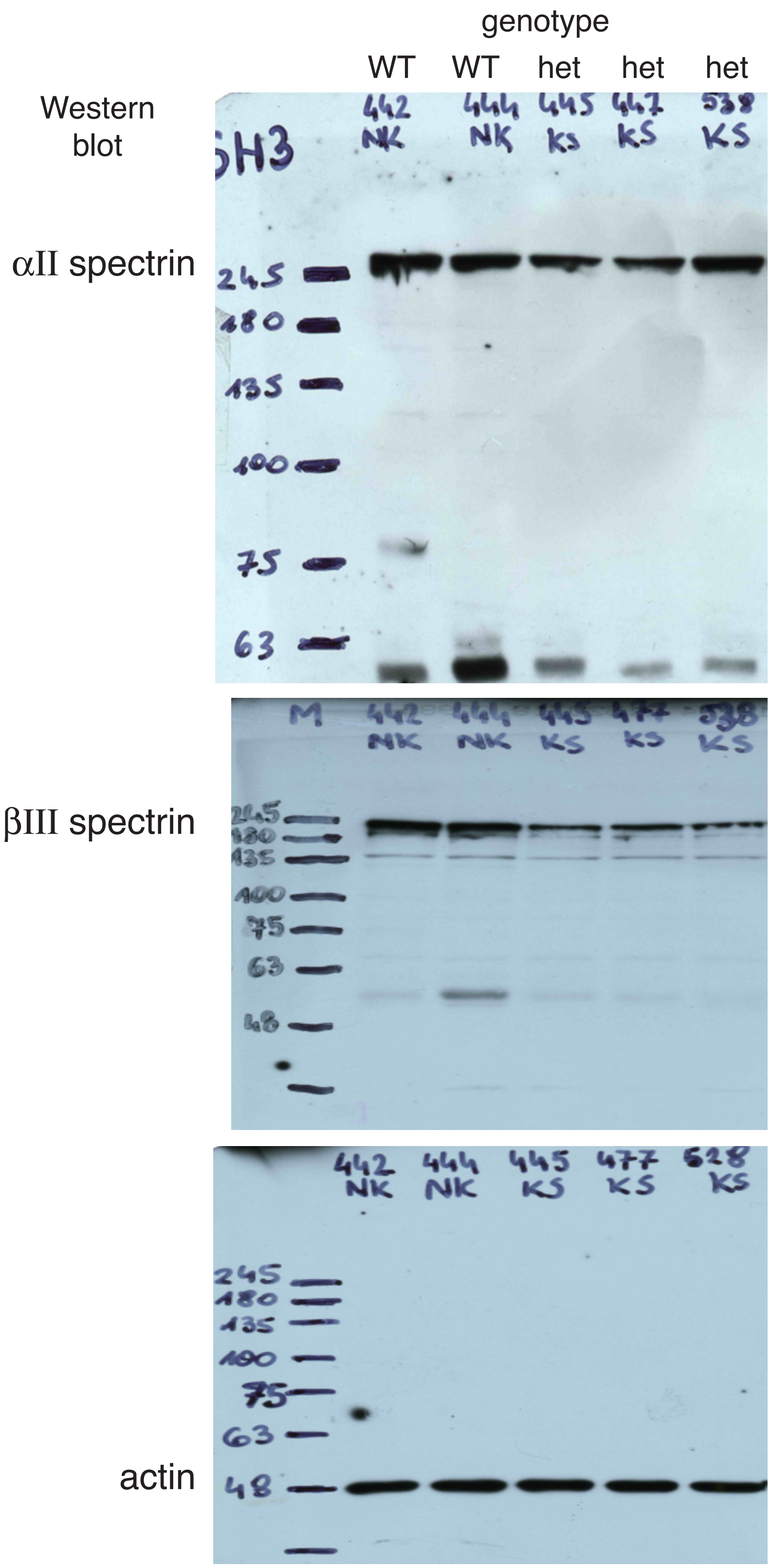

Supplemental Figure 6: Original uncropped western blots used in Figure 3.

Age-dependent ataxia and neurodegeneration caused by an αII spectrin mutation with impaired regulation of its calpain sensitivity

Arkadiusz Miazek, Michał Zalas, Joanna Skrzymowska, Bryan A. Bogin, Krzysztof Grzymajło, Tomasz Goszczynski, Zachary A. Levine, Jon S. Morrow, and Michael C. Stankewich

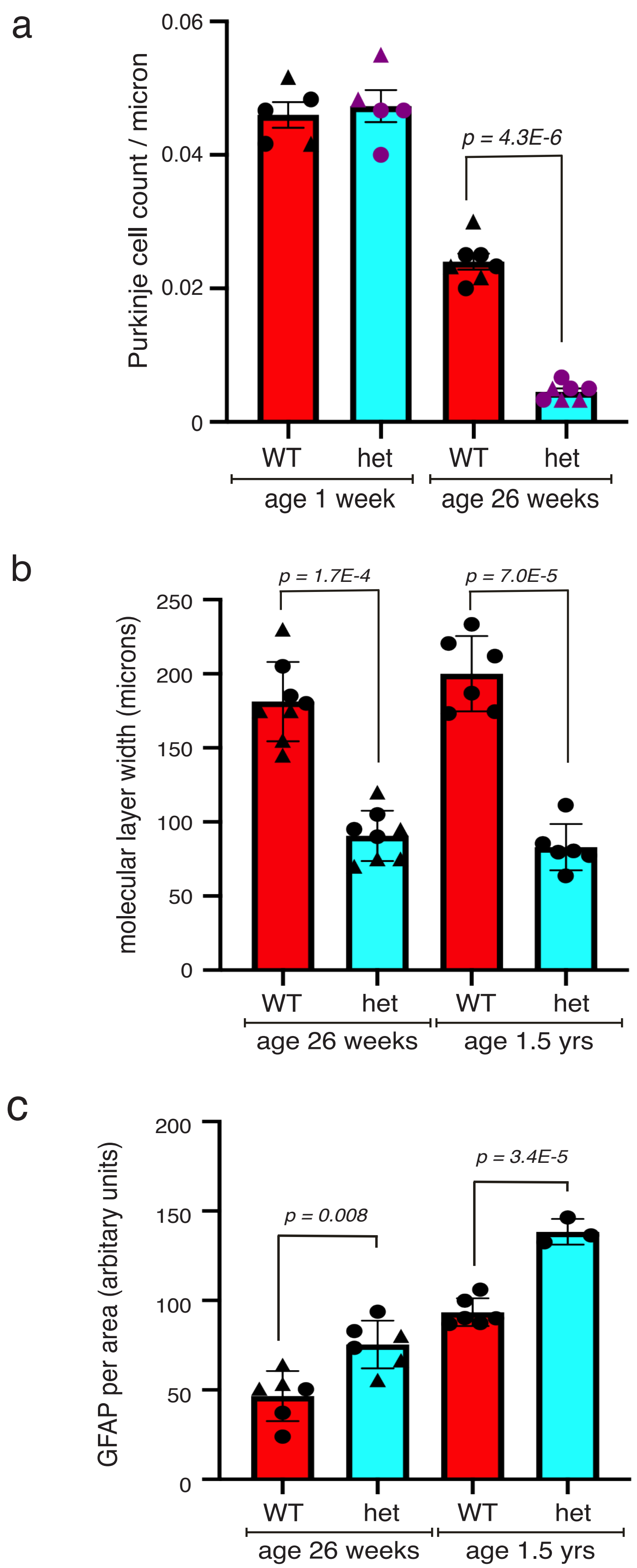

Supplemental Figure 7: Measured cerebellar changes with age.

Age-dependent ataxia and neurodegeneration caused by an αII spectrin mutation with impaired regulation of its calpain sensitivity

Arkadiusz Miazek, Michał Zalas, Joanna Skrzymowska, Bryan A. Bogin, Krzysztof Grzymajło, Tomasz Goszczynski, Zachary A. Levine, Jon S. Morrow, and Michael C. Stankewich

Age-dependent ataxia and neurodegeneration caused by an αII spectrin mutation with impaired regulation of its calpain sensitivity

Arkadiusz Miazek, Michał Zalas, Joanna Skrzymowska, Bryan A. Bogin, Krzysztof Grzymajło, Tomasz Goszczynski, Zachary A. Levine, Jon S. Morrow and Michael C. Stankewich

Supplemental Table 1: Variants identified by WES in ataxis C57/B/6J mice but not in WT littermates.

Ch	gene	name	Putative function/association	SIFT
1	AC133103.1	BAC clone, not characterized		tolerated
1	AC168977.1	BAC clone, not characterized		deleterious
1	Hjurp	HJURP Holliday junction recognition protein	DNA binding; cellular component organization	tolerated
1	Mroh2a	MROH2A maestro heat like repeat family	uncharacterized	tolerated
1	Prg4	PRG4 proteoglycan 4	proteoglycan synthesis in cartilage	tolerated
1	Rrs1	RRS1 ribosome biogenesis regulator 1	protein synthesis	deleterious
1	Ugt1a1	UGT1A1 UDP glucuronosyltransferase family 1 member A1	Crigler-Najjar syndromes - metabolism of bilirubin	tolerated
2	Sptan1	Spectrin non-erythroid alpha1	West syndrome et al.	deleterious
3	Pias3	protein inhibitor of activated STAT 3	transferase; retinal phenotype	deleterious
3	Rptn	RPTN repetin	Diminished serum repetin levels in patients with schizophrenia and bipolar disorder	tolerated
4	Zfp990	Zfp990 zinc finger protein 990	transcriptional regulator	tolerated
5	Gm17019	predicted gene, not characterized		deleterious
5	Tacc3	TACC3 transforming acidic coiled-coil	motor spindle protein that may play a role in	tolerated
6	Peg10	PEG10 paternally expressed 10	role in cell proliferation, differentiation and	deleterious
6	Phf14	PHF14 PHD finger protein 14	mitosis regulation and tumorgenesis	deleterious
7	Zfp936	Zfp936 zinc finger protein 936	transcription factor, low expression in brain	deleterious
8	Pkd1l3	PKD1L3 polycystin 1 like 3, transient receptor	cation channel pores	tolerated
8	Thap1	THAP1 THAP domain containing 1	proapoptotic factor that links PAWR to PML nuclear bodies.	deleterious
10	Duxf3	Duxf3 double homeobox family member 3	DNA binding	tolerated
11	Gm11555	predicted gene, not characterized		tolerated
12	Gm2022	predicted gene, not characterized		tolerated
13	Nkapl	NKAPL NFKB activating protein like	polymorphic associatoin with schizophrenia	deleterious
14	Gm21103	predicted gene, not characterized		tolerated
15	Krt78	KRT78 keratin 78	structural protein in epithelial	tolerated
16	Muc13	MUC13 mucin 13, cell surface associated	epithelial mucin	tolerated
16	Muc4	MUC4 mucin 4, cell surface associated	epithelial mucin	tolerated
17	Prdm9	PRDM9 PR/SET domain 9	histone methyltransferase activity that catalyzes histone H3 lysine 4 trimethylation (H3K4me3)	deleterious
17	Tbl3	TBL3 transducin beta like 3	WD40 repeat protein mediating protein-protein	tolerated
17	Vmn2r114	Vmn2r114 vomeronasal 2, receptor 114	pheromone receptor	deleterious
17	Vmn2r115	Vmn2r115 vomeronasal 2, receptor 115	pheromone receptor	deleterious
17	Vmn2r117	Vmn2r115 vomeronasal 2, receptor 117	pheromone receptor	tolerated
**x**	Cdr1	CDR1 cerebellar degeneration related protein 1	Cerebellar degeneration-related autoantigen 1	tolerated
**x**	Vmn2r121	Vmn2r121 vomeronasal 2, receptor 121	pheromone receptor	deleterious
**y**	Gm4064	predicted gene, not characterized		tolerated
